# The critical role of QM/MM X-ray refinement and accurate tautomer/protomer determination in structure-based drug design

**DOI:** 10.1007/s10822-020-00354-6

**Published:** 2020-10-27

**Authors:** Oleg Y. Borbulevych, Roger I. Martin, Lance M. Westerhoff

**Affiliations:** QuantumBio Inc, 2790 West College Ave, Suite 900, State College, PA 16801 USA

**Keywords:** CSAR set, X-ray crystallography, Quantum mechanics X-ray refinement, Ligand strain, high throughput crystallography, protonation states, tautomers, difference density Z-score, Structure guided drug discovery, Structure-based drug discovery

## Abstract

Conventional protein:ligand crystallographic refinement uses stereochemistry restraints coupled with a rudimentary energy functional to ensure the correct geometry of the model of the macromolecule—along with any bound ligand(s)—within the context of the experimental, X-ray density. These methods generally lack explicit terms for electrostatics, polarization, dispersion, hydrogen bonds, and other key interactions, and instead they use pre-determined parameters (e.g. bond lengths, angles, and torsions) to drive structural refinement. In order to address this deficiency and obtain a more complete and ultimately more accurate structure, we have developed an automated approach for macromolecular refinement based on a two layer, QM/MM (ONIOM) scheme as implemented within our DivCon Discovery Suite and "plugged in" to two mainstream crystallographic packages: PHENIX and BUSTER. This implementation is able to use one or more region layer(s), which is(are) characterized using linear-scaling, semi-empirical quantum mechanics, followed by a system layer which includes the balance of the model and which is described using a molecular mechanics functional. In this work, we applied our Phenix/DivCon refinement method—coupled with our XModeScore method for experimental tautomer/protomer state determination—to the characterization of structure sets relevant to structure-based drug design (SBDD). We then use these newly refined structures to show the impact of QM/MM X-ray refined structure on our understanding of function by exploring the influence of these improved structures on protein:ligand binding affinity prediction (and we likewise show how we use post-refinement scoring outliers to inform subsequent X-ray crystallographic efforts). Through this endeavor, we demonstrate a computational chemistry ↔ structural biology (X-ray crystallography) "feedback loop" which has utility in industrial and academic pharmaceutical research as well as other allied fields.

**Electronic supplementary material:**

The online version of this article (10.1007/s10822-020-00354-6) contains supplementary material, which is available to authorized users.

## Introduction

Thanks in large part to their speed and lower cost, virtual screening, docking, and scoring have become integral to the drug discovery process as these methods have become critical tools in the structure based drug design (SBDD) toolbox [1–8]. Unfortunately, these methods are often unable to correctly capture and sample structural water [9–11], tautomeric states [12, 13], and conformational strain [14], which leads to problems with binding mode and binding affinity prediction [15–20]. Furthermore, these problems are compounded with scoring function errors [21, 22] and inaccurate protein:ligand complex structure determination [23–25], which together negatively impact their performance in industrial drug discovery efforts. In this paper, we demonstrate a computational chemistry ↔ structural biology (X-ray crystallography) feedback loop which uses a physics-based score function as an indicator of problems in experimental structure, and we show how we can use improved refinement methods to address these structural problems (and vice versa).

X-ray crystallography is a ubiquitous technique which is used throughout the SBDD process to determine the three-dimensional (3D) atomic structure of biomolecular systems which drive lead optimization and drug design. It is the quality of these structural models which often dictates the success of high-throughput screening, docking, and scoring (e.g. rank ordering) of candidate drug molecules and subsequent lead optimization and rational drug design. Due to advances in data collection, processing, structure solution, and refinement automation, X-ray crystallography has become relatively routine in the pharmaceutical space. Unfortunately even with these advances, surveys have shown that protein X-ray models are still found to have significant atomic coordinate uncertainties and other structural errors which impact their use in pharmaceutical research [26, 27]. In particular, amino acid, ligand, and fragment R-groups containing amides, rings, and other similarly "flippable" species are particularly susceptible to uncertainties in the placement since light elements (e.g. nitrogen, oxygen, and carbon) are typically indistinguishable in macromolecular X-ray crystallography. Those structural errors have been known to negatively impact ligand binding affinity prediction [26] and X-ray model quality impacts the overall success of the SBDD effort [25, 28–30]. In addition to limitations in the X-ray experimental, conventional stereochemical-restraints used in traditional X-ray model refinement are highly approximate in that they do not account for interactions such as electrostatics, hydrogen bonds, dispersion, charge transfer, and polarization [31–33]. Moreover, these restraints consist of a detailed description of the *unbound* molecular geometry for each ligand in the structure provided in the form of Crystallographic Information File (CIF). Unfortunately, creation of *accurate* CIF’s is a nontrivial task and often their use leads to inaccuracies in *bound* ligand structures [32] due to poor or incomplete a priori understanding of in situ bound bond lengths and angles which arises from the absence of intermolecular interactions in conventional X-ray refinement functionals [29, 34–36]. Ultimately, conventionally refined structures are subject to the principle "garbage in/garbage out" and an incorrect ligand description (e.g. CIF) often leads to an inaccurate final geometry [32].

In order to address this limitation and generate X-ray structures which are better prepared for use in SBDD protocols, in previous work, we built on our DivCon, linear-scaling, semiempirical quantum mechanics (SE-QM) [37–39] package and we introduced an automated region quantum mechanics (QM) refinement technique which replaces conventional stereochemical-restraints—both for the ligand(s) and for the surrounding active site(s)—with a far more complete SE-QM based energy functional "in real time" during refinement process [36, 39]. Outside of this (these) QM region(s), protein receptor residues and non-active site waters are treated with a molecular mechanics (MM) potential leading to a single QM/MM Hamiltonian, based on the ONIOM formalism [40], which is applied across the entire structure [35]. Together, this Hamiltonian is able to capture critical intra-molecular and inter-molecular interactions, including dispersion, hydrogen bonds, electrostatics, polarization, charge transfer, metal coordination [3, 7, 8, 41, 42], which are neglected in conventional X-ray crystallographic refinement workflows. With this protocol in place, DivCon-based X-ray refinement explicitly disregards any (potentially flawed) information provided by CIF yielding more accurate ligand and active site geometry. Specifically, we have demonstrated that our DivCon, QM/MM refinement applied to the Astex Diverse Set [17] yields significant improvement not only for ligand structure but also for the entire protein:ligand complex structure [35].

In the present work, we applied this QM/MM refinement protocol to the set of structures from the Community Structure Activity Resource (CSAR) data set originally released in 2012 [43]. The CSAR set is a well curated set which includes carefully determined experimental binding affinities and which was specifically developed for the purpose of providing structures to improve available docking/scoring functions. For that reason, we chose the set to explore how our QM/MM refinement method enhances the quality of the protein:ligand geometry and how these improvements in quality also impact our ability to use physics-based functions to predict binding affinity. Furthermore, we are able to demonstrate that we can use binding affinity prediction outliers that remain even after Phenix/DivCon (QM/MM) refinement to indicate those cases which we can improve with subsequent analysis and manual, experimental density driven manipulation. Finally, in order to explore the impact of tautomer/protomer states on binding affinity prediction, we applied our XModeScore [44, 45] method to the set. This method employs the same QM/MM X-ray refinement discussed above and couples it with rigorous experimental density analysis to determine the correct protonation states (or modes) of residues and bound ligands and fragments. We hope that these enhanced structures, available in the Supplementary Information, aid in the development of next generation docking/scoring functions.

## Materials and methods

### Structure preparation and refinement

The 55 X-ray coordinate and structure factor files corresponding to five different kinase targets in the 2012 CSAR set were downloaded from the Protein Database (PDB). This set, listed in detail in Table 1 and Table S1, consists of cyclin-dependent kinase 2 (CDK2) with 15 ligands, checkpoint kinase 1 (CHK1) with 16 ligands, mitogen-activated protein kinase 1 (ERK2) with 12 ligands, urokinase-type plasminogen activator (uPA) with 7 ligands, and spleen tyrosine kinase (Syk) with 5 ligands. Hydrogen atoms were added to protein residues, water molecules, and ligands using Protonate3D [46] as implemented in the Molecular Operating Environment (MOE) v2019.0102 package from Chemical Computing Group, Inc.[47]. The default Protonate3D settings of pH, temperature, and ion concentration (Salt) of 7, 300 K, and 0.1 mol/L respectively were selected and all atoms were allowed to "Flip" so some HIS, ASN, and GLN residues may have flipped during the protonation process (see Supplementary Information for all prepared structures used in this paper). In cases with residues with alternative conformations, the default MOE protocol is to maintain the conformation ‘A’ and remove all other alternative states and this protocol was also used in the current structure preparation. It is important to note that original published CIF information was used during ligand protonation only if the CIF a) is available for the relevant ligand within the library provided in the PHENIX package, and b) passes a heavy atom graph match and other integrity checks adopted by MOE. Finally, each structure was crystallographically refined using the ONIOM QM/MM method incorporated into PHENIX package [31] as described in our previous work [35, 36].

**Table 1:** Ligand Strain energies and ZDD values, as well as Molprobity statistics for 55 CSAR structures after QM/MM ONIOM and conventional PHENIX refinements (See Table S1 for corresponding results obtained from original, published PDB files)

Phenix/DivCon (QM/MM)					PHENIX				
Strain Energy	ZDD	GBVI/WSA	Clash Score	MolProbity Score	Strain Energy	ZDD	GBVI/WSA	Clash Score	MolProbity Score
2.57	7.29	− 5.83	0.21	0.58	9.63	9.42	− 5.57	1.47	0.94
3.52	13.19	− 6.78	0.87	0.77	10.65	19.02	− 6.54	2.61	1.05
4.06	4.81	− 7.08	0.87	0.77	20.43	12.98	− 7.27	1.52	0.96
5.5	2.41	− 7.80	1.32	0.91	19.24	3.07	− 7.83	1.54	0.9
3.24	6.51	− 6.61	0.22	0.58	13.99	7.26	− 6.40	1.08	0.81
4.95	2.21	− 7.63	0.85	0.76	15.74	2.23	− 7.80	1.49	0.91
17.5	3.80	− 7.56	0.88	0.98	43.34	15.87	− 7.08	2.43	1.02
16.85	5.65	− 8.33	0.65	0.71	58.43	3.26	− 8.49	1.96	0.96
10.87	2.93	− 8.28	0.66	0.76	34.49	14.06	− 6.56	1.97	0.96
13.51	3.86	− 7.50	1.37	1.08	27.72	16.63	− 5.70	2.74	1.27
13.12	4.59	− 7.85	0.83	0.78	19.29	13.75	− 8.03	1.66	0.93
15.44	5.60	− 9.07	0.88	0.77	19.61	12.68	− 8.44	1.99	0.97
5.33	2.72	− 7.25	0.42	0.65	19.43	1.02	− 6.99	1.27	0.85
6.49	2.44	− 6.76	0.43	0.65	17.74	1.32	− 6.85	1.09	0.81
4.96	4.27	− 7.88	0.65	0.71	96.54	16.19	− 7.15	2.18	0.99
11.59	1.55	− 7.74	0.72	1	19.25	3.39	− 6.02	0.97	1.05
5.72	2.71	− 7.66	0.48	0.89	17.20	1.66	− 7.63	1.43	1.1
10.87	1.95	− 8.90	1.72	1.33	17.51	4.50	− 8.85	1.96	0.96
6.15	1.84	− 7.73	1.22	1.07	16.42	2.28	− 8.00	1.22	1.11
10.4	4.09	− 7.34	0.72	0.99	35.30	6.68	− 7.56	1.43	1.06
10.6	0.51	− 6.09	0.69	1.17	32.33	0.52	− 6.34	1.38	1
17.72	3.69	− 7.57	0.46	0.79	22.18	5.70	− 7.84	1.39	1
8.11	1.35	− 6.43	0.23	0.94	98.96	22.73	− 4.74	1.62	1.1
10.05	3.60	− 8.47	0.23	0.76	28.09	3.55	− 8.62	1.39	1.04
6.16	1.34	− 6.70	0.95	0.97	13.63	1.24	− 7.15	2.14	1.12
10.97	0.76	− 7.22	0.47	1.01	23.77	19.39	− 6.39	1.86	1.17
11.96	0.74	− 7.17	1.43	1.01	30.83	14.44	− 5.27	1.43	0.95
4.82	2.12	− 7.40	0.46	0.79	22.92	5.74	− 7.08	1.16	1.01
4.66	0.83	− 7.46	0.71	1.16	25.10	9.35	− 7.22	1.89	1.09
7.63	1.96	− 5.57	1.43	1.29	13.39	25.23	− 1.84	1.19	1
4.69	2.74	− 6.88	1.18	1.18	21.29	4.89	− 6.88	2.12	1.12
8.69	4.08	− 6.37	0.18	0.7	24.48	4.63	− 6.92	1.61	1.04
5.3	1.28	− 5.72	1.29	0.92	12.62	2.19	− 8.26	1.47	1.01
15.27	1.01	− 5.57	0.53	0.89	24.00	1.71	− 6.58	1.95	1.14
11.51	2.90	− 7.60	0.53	0.76	21.88	4.01	− 7.85	0.71	0.81
35.15	1.20	− 7.46	0.88	1	33.85	2.12	− 7.60	1.41	1.08
19.6	8.70	− 8.28	0.88	1.03	31.95	12.47	− 8.70	1.58	1.16
14.82	2.74	− 7.64	0.76	0.96	40.27	3.46	− 7.60	1.32	1.14
14.45	1.08	− 8.77	0.18	0.79	29.24	2.73	− 8.75	0.74	0.94
20.37	1.00	− 9.65	0.18	0.77	42.57	0.76	− 9.67	0.92	0.99
23.49	2.91	− 9.90	1.06	0.89	97.52	3.50	− 10.45	1.41	0.9
19.08	2.76	− 7.97	0.37	0.67	47.25	2.24	− 8.12	1.46	0.92
3.92	2.22	− 7.63	0	0.5	17.30	2.18	− 7.89	0.55	0.73
7.2	5.52	− 9.22	0.69	0.95	25.00	4.42	− 9.37	1.14	1.14
6.19	4.44	− 8.58	0.46	0.93	26.67	4.13	− 8.83	0.69	0.95
6.75	6.10	− 8.11	1.13	0.95	23.14	5.45	− 8.71	1.36	1.04
7.04	4.30	− 8.64	1.84	1.08	25.27	2.90	− 8.71	2.75	1.2
33.4	6.86	− 8.75	0.91	1.15	64.67	6.41	− 8.99	1.37	1.13
8.23	1.88	− 5.53	0.51	0.83	15.87	1.54	− 5.55	1.53	0.99
7.94	2.07	− 7.45	0.51	0.77	17.95	3.52	− 7.49	2.03	1.07
9.44	2.02	− 6.45	1.04	1.18	20.59	1.72	− 6.70	2.59	1.49
3.69	0.63	− 5.52	0.26	0.8	16.47	2.97	− 5.56	0.78	0.95
5.42	2.21	− 7.11	0	0.59	13.37	1.50	− 7.21	0.76	0.84
7.56	1.09	− 6.30	0.76	0.84	16.56	0.78	− 6.55	3.31	1.13
10.52	4.42	− 7.30	0.5	0.77	16.25	2.94	− 7.51	0.75	0.83

**Fig. 1:**
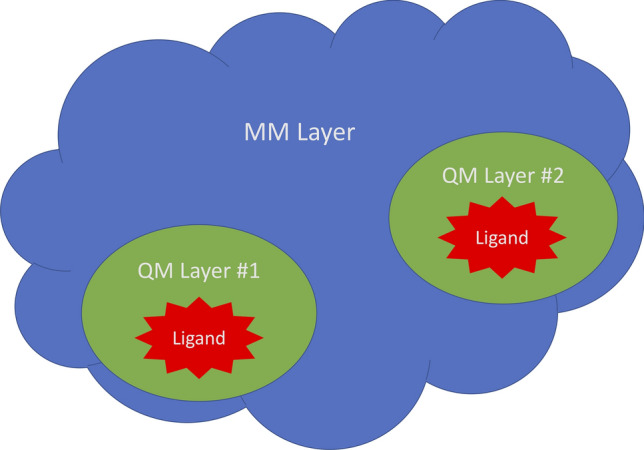
Schematic view of the QM/MM two-layer (ONIOM) concept depicting two ligand QM regions with the balance of the receptor treated as a MM layer. This method can support any number of QM regions and may even treat the entire structure as a QM structure

Briefly, Phenix/DivCon employs an automated two-layer QM/MM calculation as depicted schematically in Fig. 1. With this approach, any ligand(s) along with any surrounding active site residues are treated using the PM6 semiempirical QM Hamiltonian [48, 49] and the rest of the protein is described with AMBERff14 MM forcefield [50]—both as implemented in our DivCon Discovery Suite v.DEV-671 [39]. The two layer ONIOM QM/MM energy is computed using the subtractive scheme according to the following equation [35, 40],

1 $${\text{E}}_{ONIOM}^{QM/MM} = {\text{E}}_{region}^{QM} + {\text{E}}_{all}^{MM} - {\text{E}}_{region}^{MM}$$

where ${E}_{all}^{MM}$ is MM energy calculated for the entire system, ${E}_{region}^{MM}$ is the MM energy for the region, and ${E}_{region}^{QM}$ is the energy of the region computed with the QM method. The QM/MM gradients are computed using the similar expression (2).

2 $$\nabla x_{ONIOM}^{QM/MM} = \nabla x_{region}^{QM} + \nabla x_{all}^{MM} - \nabla x_{region}^{MM}$$

Finally, the overall refinement target *E*_*total*_ in PHENIX is presented as,

3 $${\text{E}}_{{{\text{total}}}} = {\text{ wcx}}_{{{\text{scale}}}} *{\text{W}}_{{{\text{Xray}}}} *{\text{ E}}_{{{\text{Xray}}}} + {\text{W}}_{{{\text{geom}}}} *{\text{ E}}_{{{\text{geom}}}}$$

where W_xray_ and W_geom_ are weights assigned X-ray data and geometry (stereochemistry) restraints respectively, and wcx_scale_ is the additional scale factor implemented in PHENIX [51]. In our work, W_geom_ is typically set to one while W_xray_ is a variable weight determined using an automatic procedure in PHENIX [52]. For QM/MM X-ray refinement the energy of stereochemical restraints E_geom_ is replaced with ${E}_{ONIOM}^{QM/MM}$ computed using Eq. (1). Gradients on each atom are derived as follows (4),

4 $$\left( {\nabla {\mathbf{x}}_{i} } \right)_{{{\text{total}}}} = \kappa *\Omega _{{{\text{Xray}}}} *\left( {\nabla {\mathbf{x}}_{i} } \right)_{{{\text{Xray}}}} + \Omega _{{{\text{geom}}}} *\nabla {\mathbf{x}}_{{ONIOM}}^{{QM/MM}}$$

where $\left( {\nabla{\mathbf{x}}_{i} } \right)_{{{\text{Xray}}}}$, is referred to X-ray density gradients and $\nabla {\mathbf{x}}_{ONIOM}^{QM/MM}$ are the ONIOM gradients determined using Eq. (2) meaning that *all* conventional-PHENIX stereochemical restraint gradients are replaced with QM/MM gradients [35].

To calculate these energies and gradients, for the MM portions of the structure, the AMBERff14 force field MM parameters were assigned automatically utilizing an automatic molecular perception algorithm [35] implemented in the DivCon Discovery Suite v.DEV.671. The QM regions on the other hand were extended to include all protein, water, and cofactor (if any) residues within 3.0 Å around ligands that are specified in Table 1 for each PDB. For those cases in which PHENIX-provided restraint libraries were missing or deficient, a fresh CIF file for each ligand or cofactor in each structure was generated using MOE. Finally, each Phenix/DivCon QM/MM refinement was conducted on each structure using DivCon Discovery Suite build-DEV.671 [39] "plugin" to the Phenix version phenix-1.17-3644 package [31, 35]. For comparison, conventional Phenix refinements were also carried out with the same input files, and identical default phenix.refine settings were chosen for both QM/MM and conventional refinements.

### Tautomer/protomer state determination

After completing the first round of X-ray crystallographic refinement using Phenix/DivCon, our XModeScore [44, 45] method was subsequently employed to determine the most likely tautomer/protomer state in the context of the experimental density and a second set of ONIOM QM/MM refinements were completed with these new states. Briefly, the XModeScore procedure utilizes two components: the post-QM/MM refinement local ligand strain energy (LLSE)—calculated in this case using the aforementioned PM6 Hamiltonian—and the Z-score of the experimental (X-ray) difference density called ZDD discussed in [53]. When LLSE and ZDD are determined for the set of tautomers/protomers (or flip-states, binding modes, etcetera), the XModeScore of the *i*-tautomer form can be calculated according to (5),

5 $$Score_{i} = - \left\{ {\frac{{{\text{ZDD}}_{i} - \mu_{{{\text{ZDD}}}} }}{{\sigma_{{{\text{ZDD}}}} }} + \frac{{{\text{LLSE}}_{i} - \mu_{{{\text{LLSE}}}} }}{{\sigma_{{{\text{LLSE}}}} }}} \right\}$$

where *m* is the mean value and *s* is the standard deviation of the corresponding array of data (ZDD or LLSE). Therefore, the protomer/tautomer with the highest *Score*_*i*_ corresponds to the tautomeric form *‘i’* that best fits both LLSE (calculated energy) and ZDD (experimental density) criteria. The details of how these two criteria are summarized below.

### Local ligand strain energy

The LLSE, as opposed to the global ligand strain, is used in XModeScore in order to measure the relative ligand strains based on the very small, localized conformational changes due changes in tautomer/protomer states, rotamer flip states, and so on. The LLSE or *E*_*Strain*_ is the difference between the energy of the protein-bound ligand conformation and the isolated ligand conformation and is computed according to the Eq. (6),

6 $$E_{Strain} = E_{SinglePoint} - \, E_{Optimized}$$

where *E*_*SinglePoint*_ is the single-point energy computed for the ligand X-ray geometry, and *E*_*Optimized*_ is the energy of the optimized ligand that or the local minimum [54]. All LLSE calculations in this project were calculated using the PM6 Hamiltonian [48, 49] as implemented in DivCon.

Ligand strain is generally thought of as a measure of how much strain the ligand must accept or accommodate in order to bind with the protein of interest. Colloquially, in our experience, we think of strain being caused by three different components: Method Induced Strain (MIS) which is the strain attributed to the refinement method itself (e.g. inaccurate CIF parameters, pair potential approximations, protein:ligand interactions, and so on); Docking Induced Strain (DIS) which is the strain associated with initial placement of the ligand within the experimental density; and Target Induced Strain (TIS) or—ideally—a minimal, naturally occurring strain caused by interactions between the protein and ligand. Often it is difficult to "tease out" which components are causing the greatest impact on the calculation of ligand strain. For example, in practice when calculating ligand strain, TIS and MIS often appear to overlap significantly: is the calculated strain naturally occurring or is it due to inaccuracies in protein:ligand pair potential and so on? Given the fact that the present study does not involve re-docking or other hand re-placement of the ligand unless otherwise indicated, the final reported strain is generally limited to the same radius of convergence of the (published) input ligand coordinates. Therefore, we did not endeavor to answer this question and instead we focused on how much an improved potential (*i.e.* QM/MM in this case) alone addresses the strain with the assumption being that any remaining strain is primarily a mixture of (naturally occurring) TIS and (artificial) DIS. *In fact, in cases where higher than expected local ligand strain is reported upon completion of QM/MM X-ray refinement, this is likely an indicator that additional sampling is warranted and underscores how these tools can be used in support of the aforementioned computational chemistry ↔ structural biology (X-ray crystallography) feedback loop.*

### Z-score of the difference density (ZDD)

In 2012, Tickle [53] described a novel quality indicator—the real-space Z-score of difference density or ZDD—in order to measure the *accuracy *of an X-ray model. ZDD is in contrast to the conventional Real Space Correlation Coefficient (RSCC) which correlates with both accuracy *and* precision of the model and is therefore often unable to measure model inaccuracy. A detailed mathematical description of ZDD can be found in [44, 53], but briefly, the Z-score for a point difference density value is expressed by Eq. (7),

7 $$Z\left( {\Delta \rho \left( {\varvec{r}} \right)} \right) = \frac{{\Delta \rho \left( {\varvec{r}} \right)}}{{\sigma \left( {\Delta \rho \left( {\varvec{r}} \right)} \right)}}$$

where $\sigma \left(\Delta \rho \left({\varvec{r}}\right)\right)$ is the standard deviation of the difference density and corresponds to the random error of the model and is pure *precision*, while the Z-score of the difference density is a measure of the residual, non-random error and is pure *accuracy*. In order to limit the noise found in the final value, we assume that the difference density *Z* values should approach a normal distribution of random errors with zero mean and unit standard deviation. The presence of negative peaks or positive peaks, which significantly deviate from the expected distribution, indicates one or more problems with the model. One can then calculate the standard chi-square (**c**^2^) statistic for a subset of the negative density values and the positive density values, and find the subset of values of ${x}_{\left(i\right)}^{2}$ which maximize the probability *p*_max_ over *k*,

8 $$\begin{gathered} p_{max} = {\max}_{k} {\text{p}}\left( {{\upchi }_{k}^{2} \le \mathop \sum \limits_{i = k}^{N} x_{\left( i \right)}^{2} } \right) \hfill \\ \gg {\max}_{k } {\text{P}}\left( {{1 \mathord{\left/ {\vphantom {1 2}} \right. \kern-\nulldelimiterspace} 2}\mathop \sum \limits_{i = k}^{N} x_{\left( i \right)}^{2} ; \,\left( {N + 1 - k} \right)/2} \right) {\text{I}}\left( {2\Phi \left( {x_{\left( k \right)} } \right) - 1;\quad k - 1, \quad N + 1 - k} \right) \hfill \\ \end{gathered}$$

where the function *P* is the lower normalized gamma function representing the cumulative distribution function (CDF) of $\chi _{k}^{2}$. The second function, *I*, is also computed as the complement and this becomes the normalized incomplete beta function (CDF of a normal order statistic) [55] which accounts for the ‘multiple comparisons’ correction [56]. The ZDD is then evaluated as the two-tailed normal Z-score which corresponds to the maximal value *p*_max_ over *k* of the cumulative probability of ${\chi }_{k}^{2}$ derived from (8),

9 $${\text{ZDD}} = - F^{{ - {1}}} \left( {\left( {{1 } - p_{max} } \right)/{2}} \right)$$

where the function *F* is the CDF of the normal distribution, $2F\left(\left|Z\right|\right)-1$ is the CDF of the half-normal distribution of the absolute value of a normal variate *Z*, and *F*^−1^ is the inverse function or the value of *Z* corresponding to a given probability. Once these calculations are performed, we obtain a set of negative density values and a set of positive density values. The ZDD− corresponds to incorrectly positioned atoms while the ZDD+ is due to missing atoms. In order to calculate ZDD, the ZDD- and ZDD+ metrics are taken together as defined in (10).

10 $${\text{ZDD}} = {\max}\left( {{\text{abs}}\left( {{\text{ZDD}} - } \right),{\text{ ZDD}} + } \right)$$

Finally, with LLSE and ZDD in place, in order to calculate XModeScore, multiple tautomer/protomer states are generated and crystallographically refined (using QM/MM refinement) and the LLSE and ZDD are calculated for each state. As with the refinement settings noted above, the XModeScore jobs were based on the QM/MM X-ray refinements with the QM regions defined using a 3 Å radius.

### Calculated (predicted) protein:ligand binding affinity

To evaluate the theoretical binding affinity between each ligand and its corresponding protein target, we employed the Generalized-Born Volume Integral/Weighted Surface Area (GBVI/WSA) score function [57] as implemented in MOE2019.01. In all cases in which this score is discussed in this work, the score as calculated on each protein:ligand pose "in place" *without* performing any docking or subsequent MOE-based structure minimization. The AMBER10 potential coupled with atomic charges and ligand parameters calculated using Extended Hückel Theory (Amber10:EHT) as implemented in MOE was used for all MOE-based calculations. It is notable that the GBVI/WSA score function was chosen instead of a quantum mechanics-based score function, like QMScore [58], to demonstrate that X-ray structures determined with Phenix/DivCon (a QM/MM functional) may be used—without modification—with a classical/traditional score function. For the sake of comparison, the alternative score functions available in the MOE v2019.0102 platform were also summarized including London dG (LDG), ASE Score (ASE), Affinity dG (ADF), and Alpha HB (AHB).

### Overall crystallographic structure quality metrics: MolProbity score and Clashscore

MolProbity is included as a module in the PHENIX package and the method incorporates several model validation tools encompassing multiple quality criteria [59]. Specifically, the MolProbity score (MPScore) is a logarithm-based score which combines three key component metrics including Ramachadran plot statistics, rotamer outliers, and clashscore [60]. The lower the MPScore the better the model. The Clashscore, which is a sub-score of the MPScore, is also reported and corresponds to the number of clashes per 1000 atoms. The Clashscore is determined through nonbonded atom contacts and is calculated within the program Probe using a rolling probe algorithm [61]. A clash is counted when the Probe-generated dot surface around one atom overlaps with the dot surface surrounding another atom by an amount greater than 0.4 Å [27]. The higher the number of clashes, the more the model may be adopting a "high energy" or unlikely conformation [59].

## Results and discussion

### QM/MM vs conventional X-ray refinement of CSAR set

#### Protein (target) structure quality metrics

As shown in Supplementary Table S2, the application of the QM/MM method only insignificantly affects R-factors that measure the overall agreement between the crystal model and the experimental structure factors. For example, average R_free_ after QM/MM and conventional refinements for the CSAR set are virtually identical (0.206 ± 0.01 and 0.205 ± 0.01, respectively), and the average QM/MM R_work_ of 0.173 ± 0.008 is only marginally higher than the value after the conventional refinement (0.170 ± 0.007). This observation would suggest that there is slightly less crystallographic model overfitting in the QM driven refinements, but overall, this is congruent with our previous research [36] and it shows that the QM/MM refinement does not damage the models being considered and these models are in fact X-ray structures. The MolProbity score and clashscore are used to characterize of the overall quality of protein structures [29, 35, 59, 62], and these metrics show that the Phenix/DivCon refinement is superior and addresses clashes which the conventional refinement does not appear to address (Table 1). In particular, the clashscore of QM/MM refined structures (0.72 ± 0.23) is, on average, 2 × lower (better) than after the conventional refinement (1.56 ± 0.37). A recent survey of PDB structures [63] indicates that the average clashscore of all structures deposited after 2010 is about 5 with the range of 1–99% of all clashscore values being from 0 to 50. It is notable that in our previous work we observed a larger (4.5-fold) improvement in clashscore for the Astex set as a result of QM/MM refinement as compared to the conventional protocol [35]. Such a discrepancy can likely be attributed to the nature of these two sets. While the Astex set is a highly diverse protein set, the CSAR benchmark is a curated set which only contains 5 different protein targets bound to a variety of ligands. Furthermore, given the stated goals of the original investigators who developed the CSAR set, they may have been more cognizant of potential clashes and addressed them prior to publication (even within the confines of the original, conventional refinement process they had at their disposal).

#### Ligand structure quality metrics

**Fig. 2:**
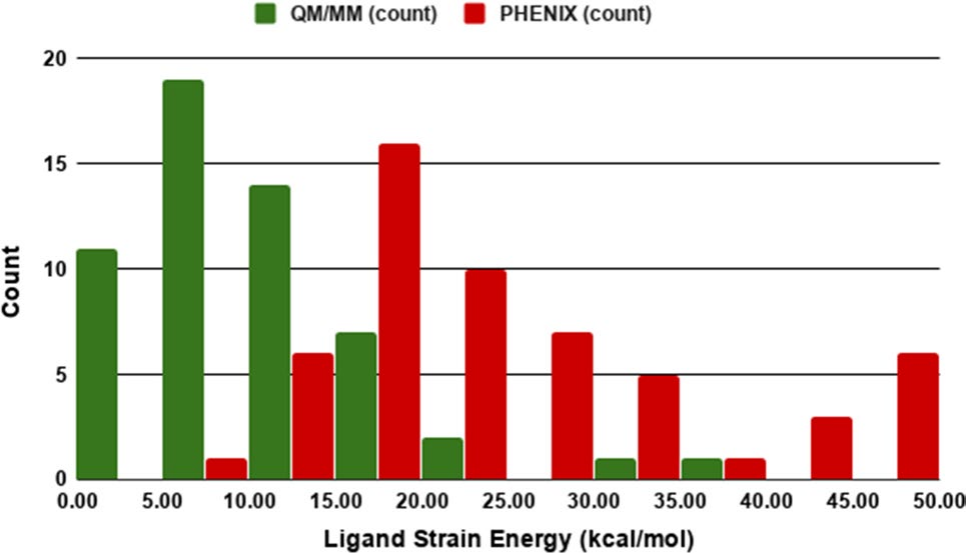
Histogram of Ligand Strain Energy (LLSE) distributions for ligands from 55 CSAR structures refined using QM/MM method and conventional PHENIX. The lower the LLSE the less strain the ligand must accommodate to fit within its associated active site

The local ligand strain method is used to explore refined ligand structural models [24, 29, 64, 65], and LLSE is used to evaluate the quality of the region refinement [35, 36, 66]. In the present study, we find that the average local ligand strain energies calculated over 55 ligands of the CSAR set after Phenix/DivCon refinement is 10.45 ± 3.28 kcal/mol and this observation is similar to the average found in our previous work (9.95 ± 3.77 kcal/mol) for the Astex set after our QM/MM refinement [35]. The average local ligand strain energy of the CSAR ligands after the conventional refinement is 28.53 ± 5.76 kcal/mol or about 2.8-fold higher than in the QM/MM Phenix/DivCon refinement. Again this finding is consistent with the previously observed average improvements in the ligand strain energy by ~ 3.5-fold after QM driven refinements in our previous studies [36]. The strain energy histogram (Fig. 2) shows a clear peak for QM/MM strain energies that covers the bins 1–3 that comprise 44 QM structures in the range from 0 to 15 kcal/mol. The strain energy distribution of PHENIX refined ligands have a peak around 20 kcal/mol with a long tail that covers the range up to 50 + kcal/mol. Given that this ~ threefold improvement in strain is solely attributable to the use of QM/MM refinement, the balance of the LLSE is likely due to a mixture of DIS and TIS and subsequent efforts could include ligand (and active site) sampling to further minimize the strain.

**Fig. 3:**
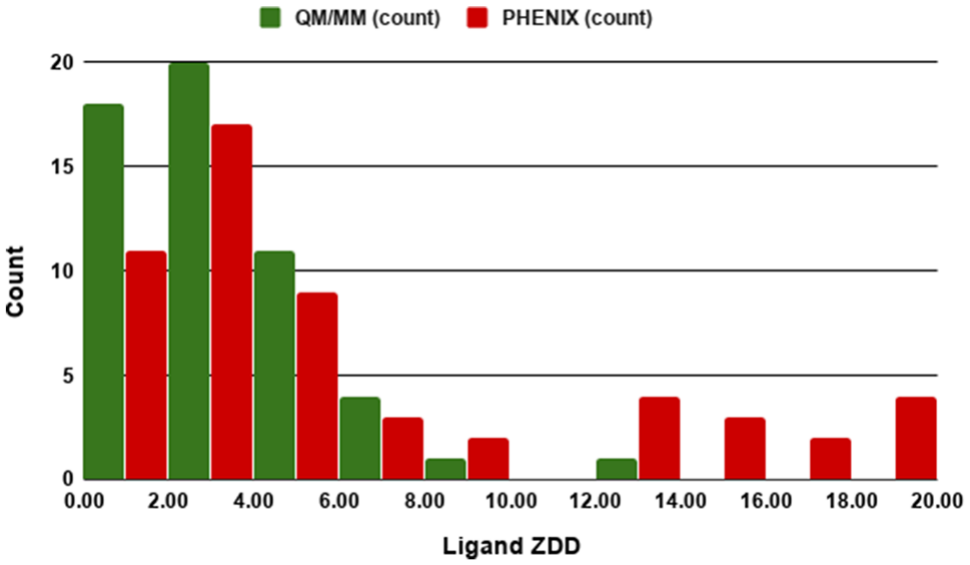
Histogram of Ligand Z-score of the difference density (ZDD) distributions for ligands from 55 CSAR structures refined using QM/MM method and conventional PHENIX. The lower the ZDD the more accurate the model versus the experimental density

**Fig. 4:**
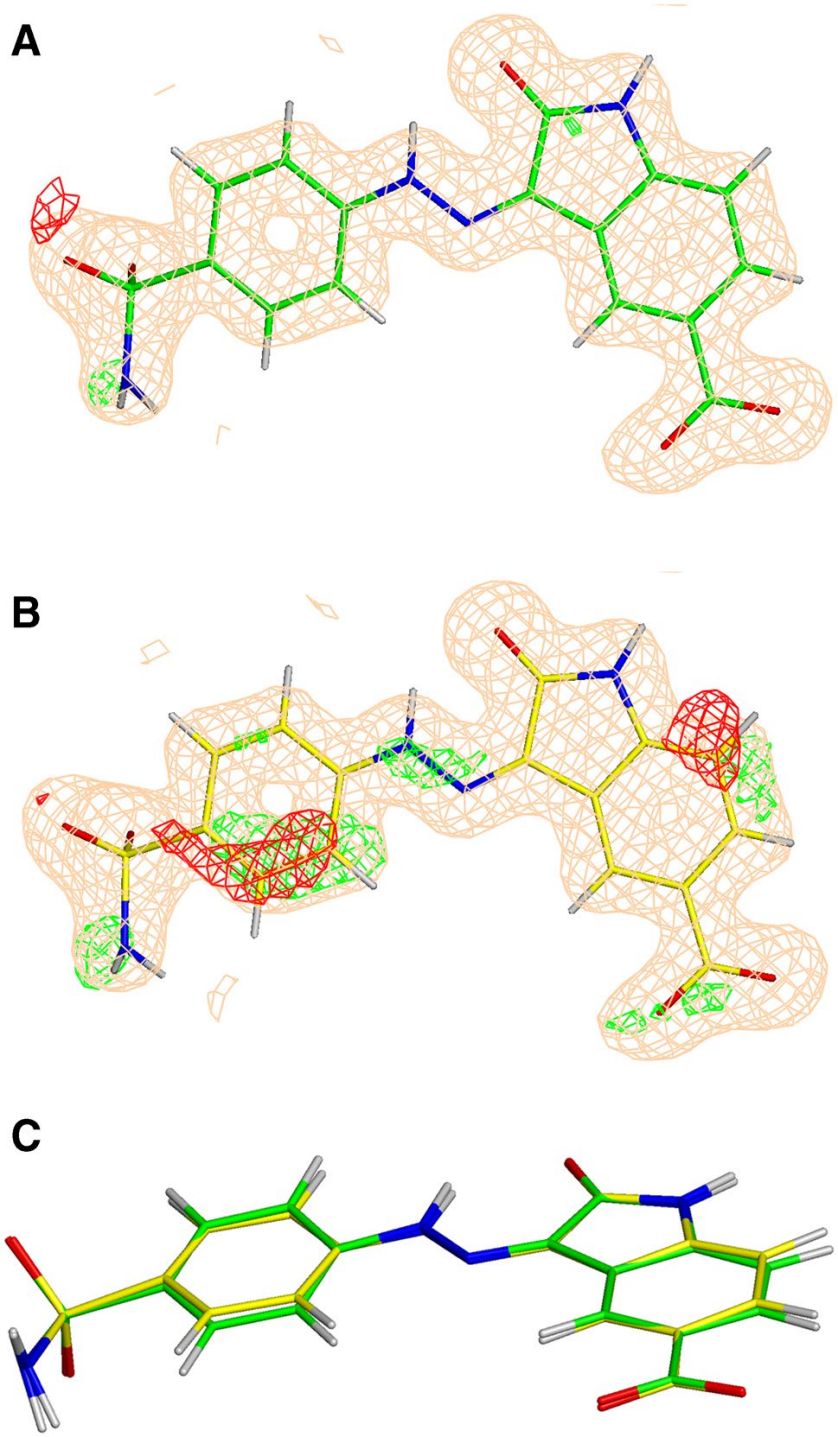
The σ_A_-weighted *mFo-DFc* difference electron density map drawn at 3σ level around the ligand (ligand ID 60 K) in the PDB structure 4FKU refined with QM/MM (**a**) and conventional (**b**). The σ_A_-weighted *2mFo-DFc* electron density map is contoured at 1 σ. **C** is provided as an overlay of the two conformations

In our previous results with the Astex Diverse Set, we showed that ligand ZDD remained essentially unchanged between QM-driven refinement and conventional refinement [35]; however, the current study with the CSAR set shows better distribution of the ligand ZDD values after the Phenix/DivCon refinement. The histogram for ZDD (Fig. 3) indicates that the population of the 1st bin (0–2 ZDD units) contains 1.5 times more QM/MM refined structures versus Phenix alone. Furthermore, the average ZDD for the ligands in QM/MM-refined structures (3.23 ± 1.37 units) is two times lower (better) than after the conventional refinement alone (6.55 ± 2.01 units). As an example, consider the refinement of CDK2 in complex with inhibitor 60 K (PDB entry 4FKU). The difference density map after the conventional refinement exhibits both positive and negative peaks around the phenyl ring of the ligand (Fig. 4b) resulting in a ZDD of 14.0. When Phenix/DivCon refinement was performed however, this process leads to an appropriate shift and rotation of the ligand such that those peaks are properly accommodated and removed by the model. As a result, the difference density peaks around the phenyl ring are not observed on the QM/MM difference map (Fig. 4a) leading to a corresponding ZDD decrease (improvement) to 4.7.

### Impact of improved refinement on binding affinity prediction

**Fig. 5:**
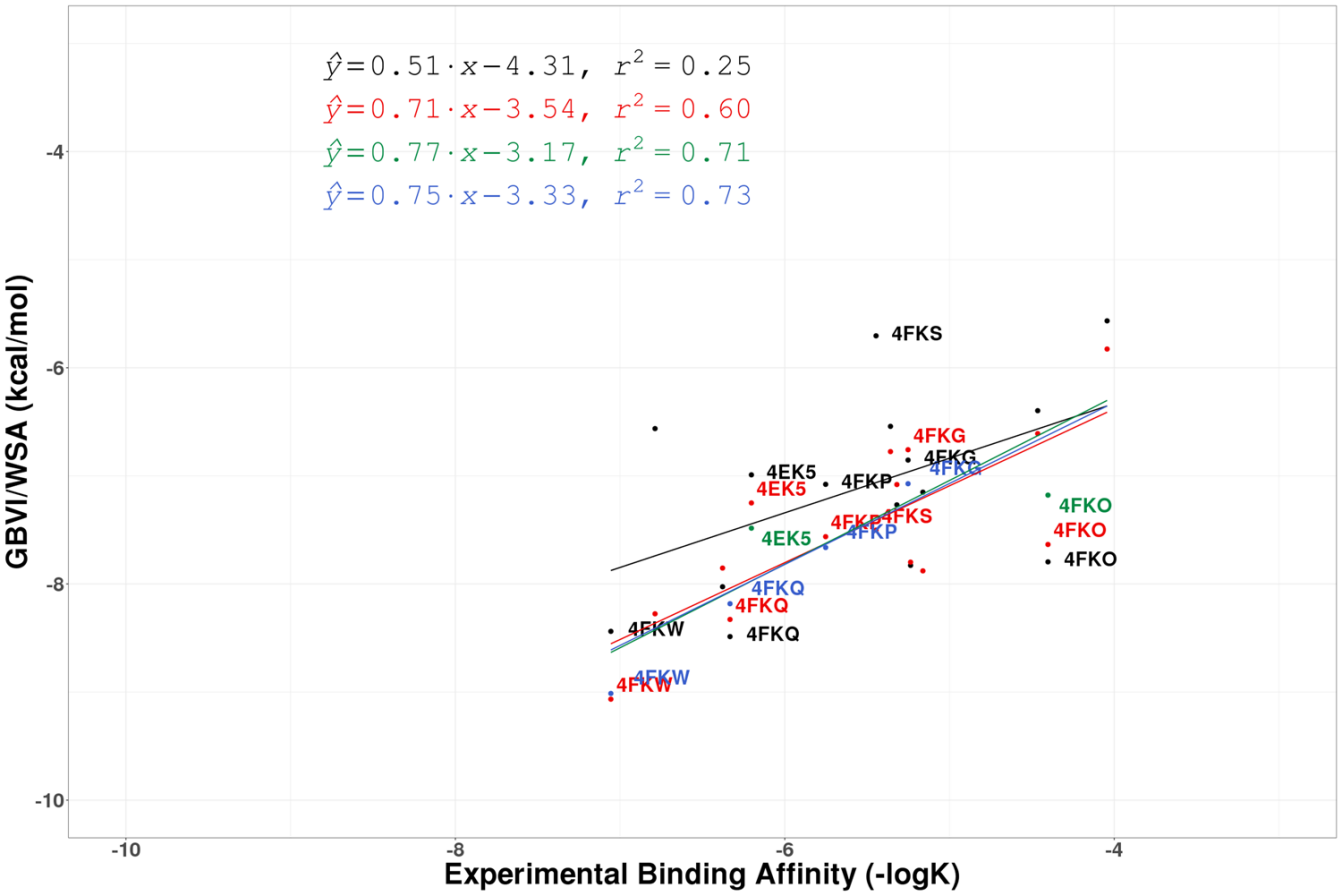
The regression lines of the correlation between experimental affinity (− logK) and computationally predicted GBVI/WSA scores for the 15 protein:ligand CDK2 complexes for PHENIX structures (*Black*), QM/MM structures (*Red*), hand-modified QM/MM structures (*Green*), and QM/MM refined structures with XModeScore chosen tautomers (*Blue*). Points involving structures discussed in the paper are labeled

**Fig. 6:**
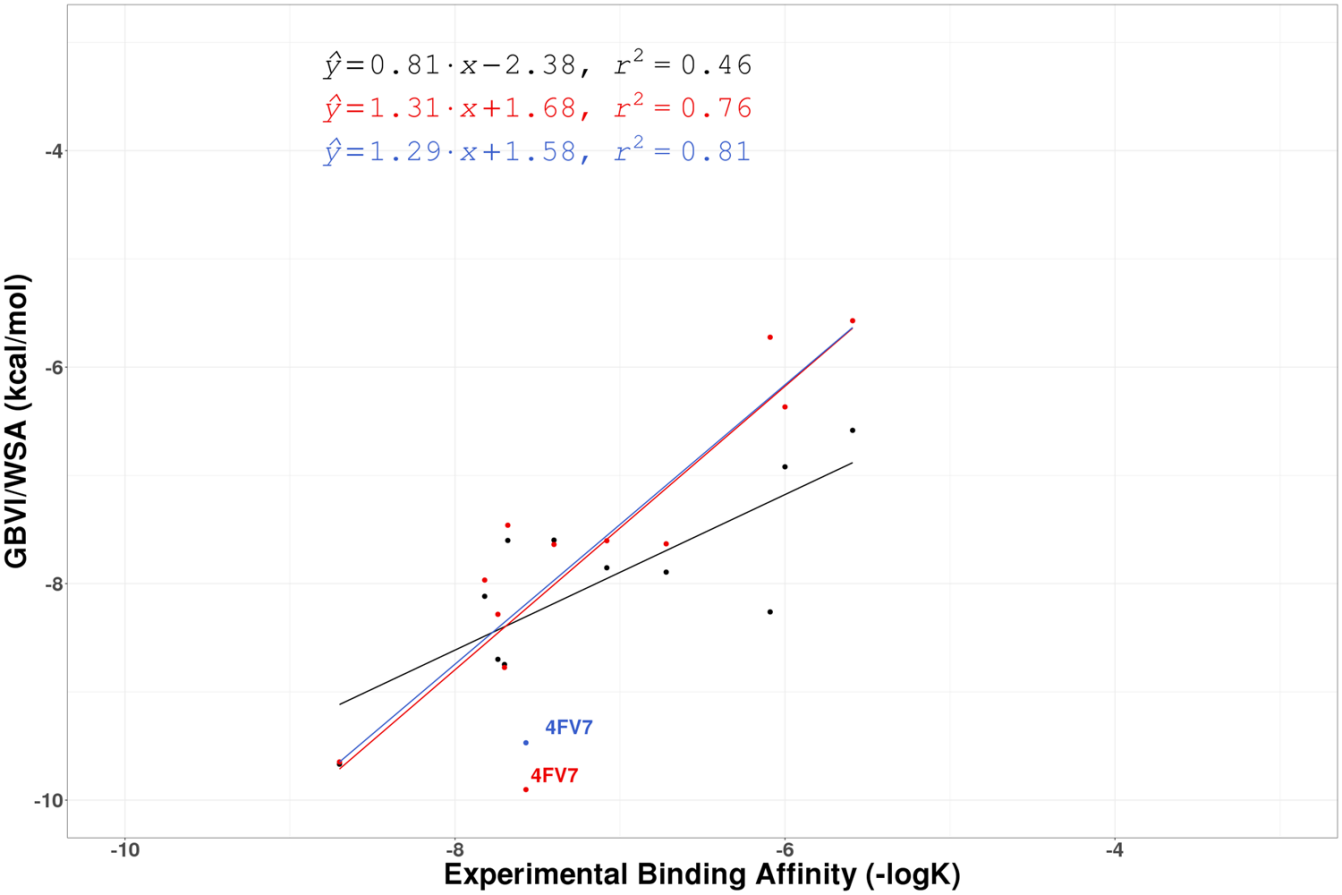
The regression lines of correlation between experimental affinity (− logK) and computationally predicted GBVI/WSA scores for the 12 protein:ligand ERK2 complexes for PHENIX structures (*Black*), QM/MM structures (*Red*), and QM/MM refined structures with XModeScore chosen tautomers (*Blue*). Points involving structures discussed in the paper are labeled

**Fig. 7:**
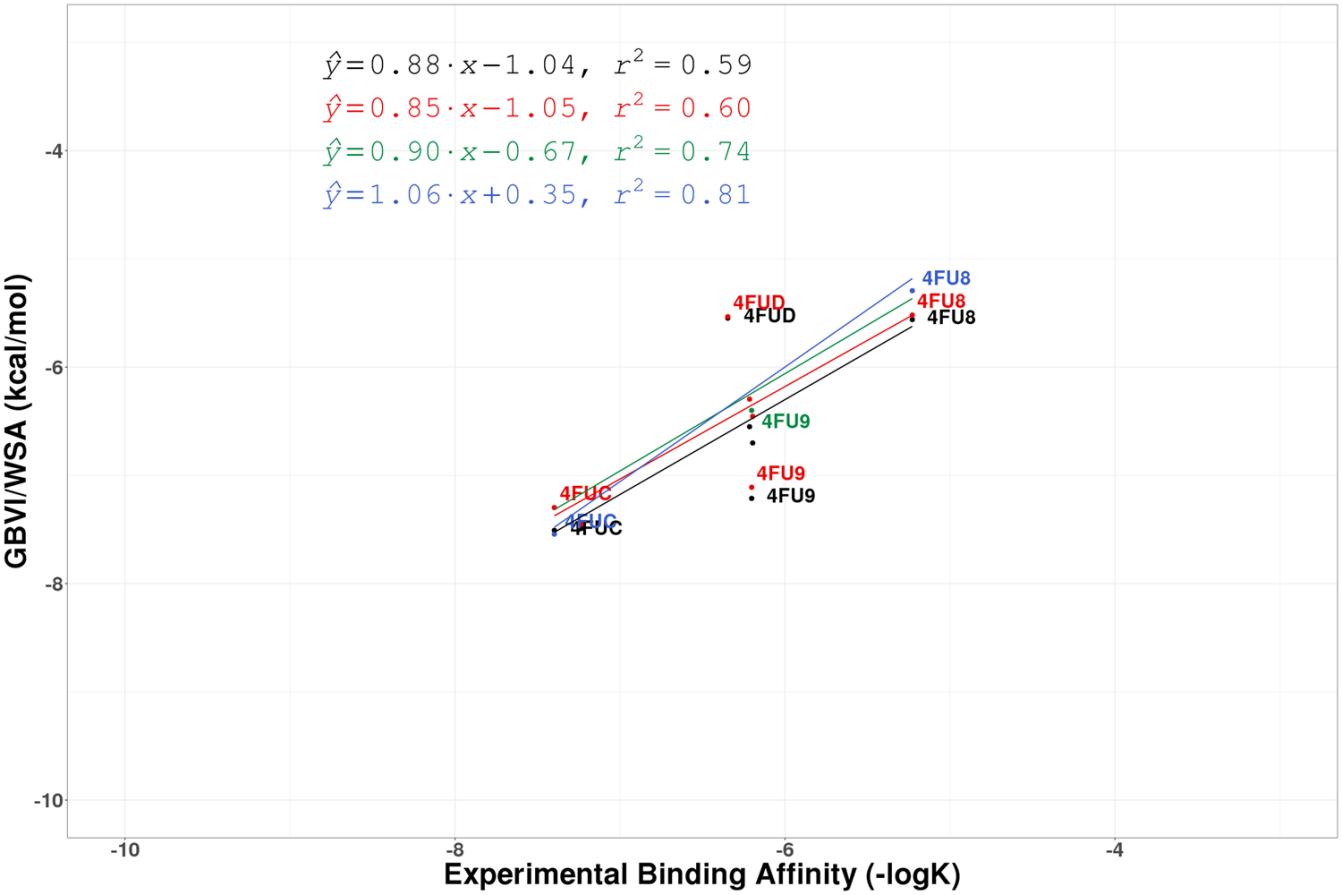
The regression lines of correlation between experimental affinity (− logK) and computationally predicted GBVI/WSA scores for the 7 protein:ligand uPA complexes for PHENIX structures (*Black*), QM/MM structures (*Red*), hand-modified QM/MM structures (*Green*), and QM/MM refined structures with XModeScore chosen tautomers (*Blue*). Points involving structures discussed in the paper are labeled

**Fig. 8:**
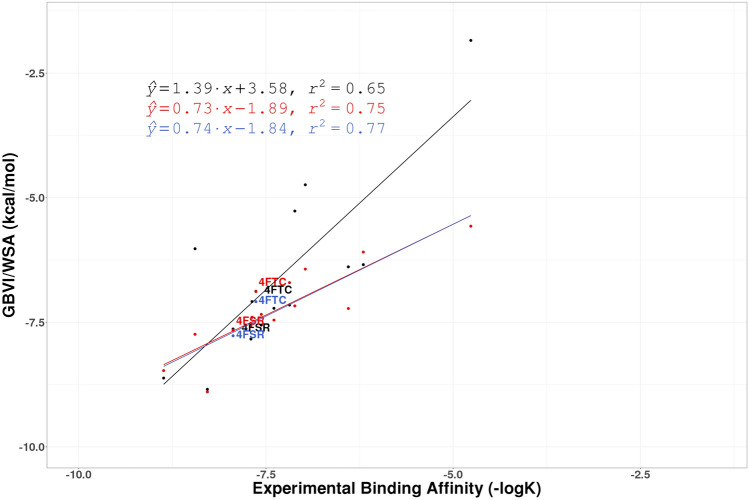
The regression lines of correlation between experimental affinity (− logK) and computationally predicted GBVI/WSA scores for the protein target CHK1 for PHENIX structures (*Black*), QM/MM structures (*Red*), and QM/MM refined structures with XModeScore chosen tautomers (*Blue*). Points involving structures discussed in the paper are labeled

**Fig. 9:**
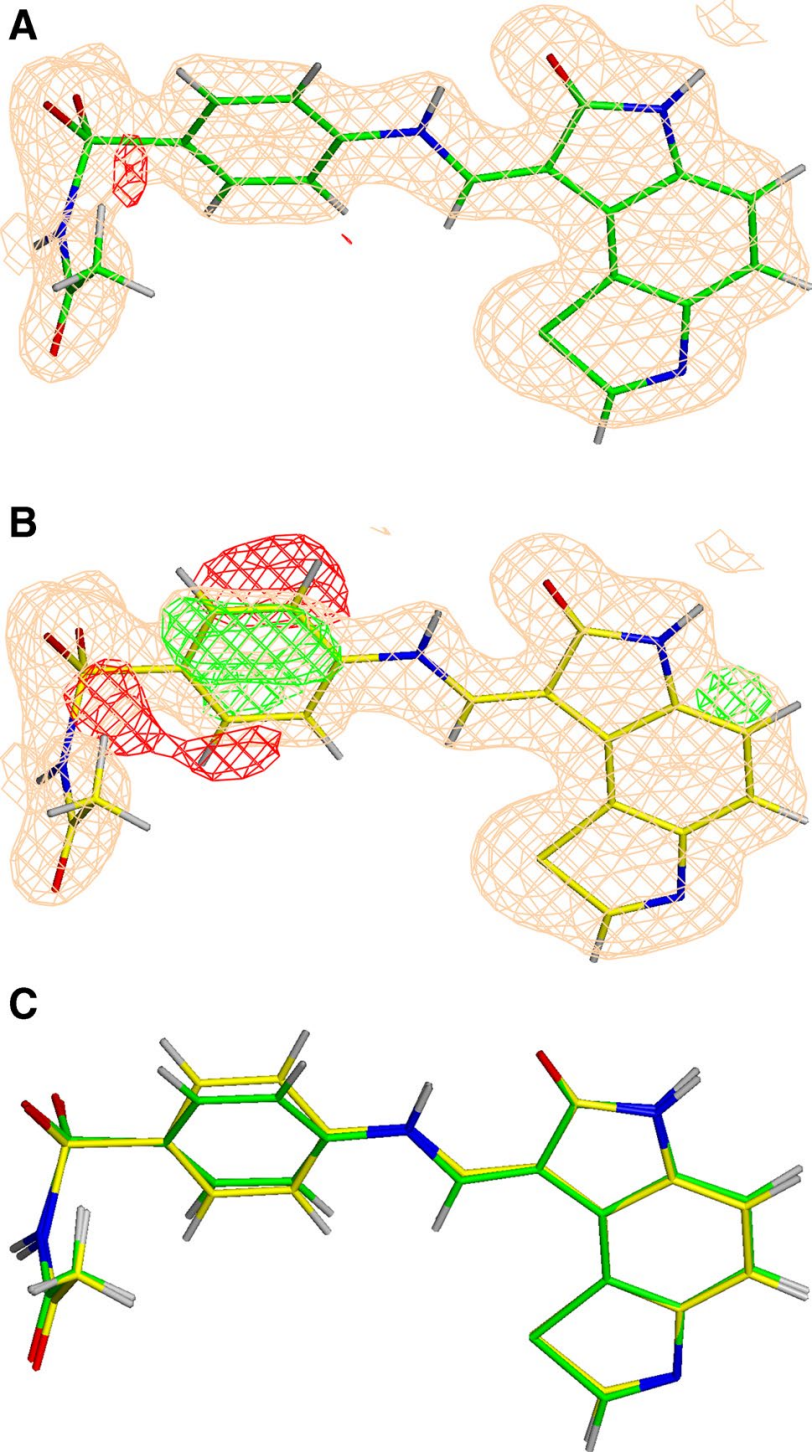
The σ_A_-weighted *mFo-DFc* difference electron density map drawn at 3σ level around the ligand (ligand ID 46 K) in the PDB structure 4FKS refined with QM/MM (*green*) (**a**) and conventional (*yellow*) (**b**), as well as the superimposition of the two structures (**c**). The σ_A_-weighted *2mFo-DFc* electron density map is contoured at 1σ

In addition to standard crystallographic and chemical metrics described above, we used the GBVI/WSA score available in MOE to evaluate the impact of these improved structures on our ability to accurately predict binding affinity. While correlating predicted binding affinities with experimental binding affinities is a trivial (if often fraught) task in SBDD, it has not generally used in the context of structure evaluation. For each of the five CSAR targets considered (CDK2, CHK1, ERK2, uPA, and Syk) and for each of the X-ray refinements performed, the correlations between experimental binding affinity (− logK) and computationally predicted GBVI/WSA scores were explored and these results are presented in Figs. 5, 6, 7, and 8 (lines/dots/equations/correlations shown in Red compared to those in Black which correspond to the QM/MM and conventional refinements respectively) and summarized in Table 1. The ERK2 and CHK1 sets exhibit the highest correlation among the CSAR proteins based on QM/MM refined structures (*Red* dots/lines on Figs. 5, 6, 7, and 8) with R^2^ ranging between 0.75 and 0.76. On the other hand, the five structures of the Syk set produce only poor correlation with the experimental values but the R^2^ (0.27) after the QM/MM refinement is still higher as compared to the conventionally refined set (0.05). Nevertheless, the Pearson correlation coefficient remains negative (− 0.51) even for the QM refined structures, and we decided not to pursue this data set any further. The R^2^ values for uPA are similar after the two types of refinements and CHK1 shows a moderate improvement when refined with QM/MM (Figs. 7 and 8). The most significant differences between Phenix/DivCon and conventionally refined structures are observed for the CDK2 and ERK2 sets (Figs. 5 and 6). For conventional structures of the CDK2 set, Fig. 5 (*Black* dots/lines) shows a scattered relationship between GBVI/WSA score and experimental binding affinity with virtually no correlation to the experimental − logK (R^2^ = 0.25). After QM/MM refinement however, the relationship yields a clear trendline with a significant R^2^ correlation of 0.60 (*Red* dots/lines on Fig. 5). The analysis of the model versus density for the 15 CDK2 structures indicates that the average ZDD (4.9 units) for QM/MM structures is 2 × lower (better) than that of the conventional structures (9.1 units). For example, these structural changes lead to improved ZDD’s for the CDK2 structure 4FKU (Fig. 4). When performing a predicted vs. experimental affinity outlier analysis, one of the worst offenders is 4FKS (Fig. 5) with the residual of 1.36. A superimposition of the refined structures indicates a different orientation of the benzyl moiety after QM/MM X-ray refinement (Fig. 9) which results in a significantly lower (better) ZDD around the ligand (3.86 units) compared to the ZDD yielded by the conventional refinement (16.63 units). This improved X-ray model leads to a decrease of GBVI/WSA score from − 5.70 to − 7.50 kcal/mol which shifts the predicted value of 4FKS practically to the trendline leading to the significantly improved correlation (0.60 versus 0.25). This improvement in binding affinity prediction is observed based on QM/MM refinement alone.

### Impact of structure modification

Up to this point in the discussion, any improvements in predicted versus experimental binding affinity correlation are attributable solely to the addition of a more complete functional (i.e. PM6/AMBERff14) to the X-ray refinement processes and no other "by hand" modification of the structures was performed. One could say that these improvements are reached within limited radius of convergence inherent to an optimization/refinement process. However, given the improved models leading to improved correlations, outliers which remain after refinement can be indicative of structural issues which can be "fed back" to the X-ray crystallography effort. Once we are sure that the structures are chemically correct within the limits of the starting model placement, outliers can often be attributed to actual structural problems in the model. We therefore used the correlations shown in Figs. 5, 6, 7, and 8 (black lines/dots for conventional refinement and red lines/dots for QM/MM refinement) and noted those cases which diverged appreciably and manually studied each case to see if there were obvious structural problems (e.g. missing bridging waters, questionable "flip" states, misplaced atom positions, and so on) in the original PDB model. For the sake of simplicity, we focused on the protein structure regions around the ligand (the active site of each model) and obvious structural defects which were clearly justified by positive/negative peaks of the difference electron density even after QM/MM refinement and we did *not* perform any further sampling (e.g. model building, simulated annealing, docking, etcetera). These cases are explored in detail in the sections below and are depicted in Figs. 5, 6, 7, and 8 (lines/dots/equations/correlations shown in Green).

#### uPA structures

**Fig. 10:**
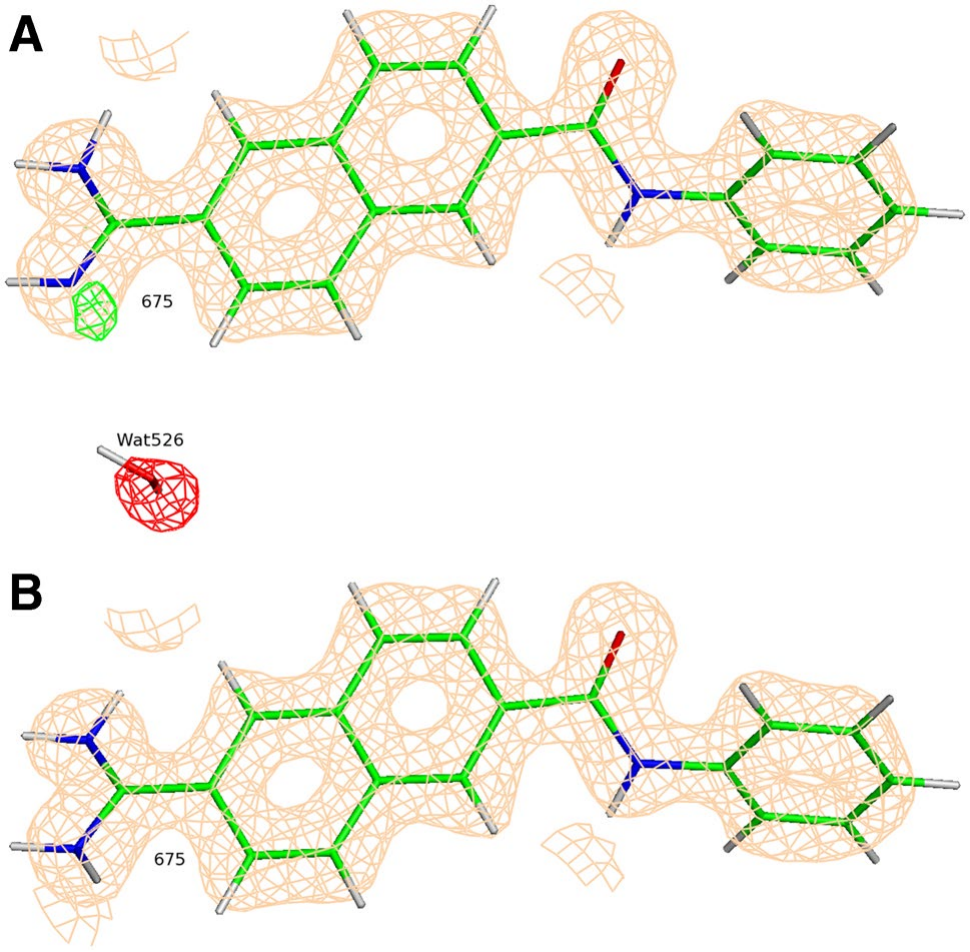
Positive (*green*) and negative (*red*) peaks of the σ_A_-weighted *mFo-DFc* difference electron density map around the ligand (ligand ID 675) and Wat526 in the binding pocket of the protein target uPA in the PDB structure 4FU9 refined with QM/MM before (**a**) and after (**b**) the manual fit. The σ_A_-weighted *2mFo-DFc* electron density map is contoured at 1 σ

An analysis of the uPA correlation plots for structures after the QM/MM refinement (Fig. 7) indicates that 4FUD and 4FU9 are the worst outliers on the graph. Unfortunately, the electron density map of the 4FUD outlier provides no clear opportunities to modify the input structure. On the other hand, as depicted in Fig. 10a, there are several questionable peaks of electron difference density observed even after the initial QM/MM refinement of 4FU9. First, the small peak of the positive difference density around the atom N18 suggests that there is an alternative protonation state of the ligand 675. This conclusion was later confirmed by XModeScore (see below). Second, the water molecule (Wat526) in the vicinity of the ligand exhibited a large peak of the negative electron density, and hence we can likely exclude this water molecule from the model. Third, the succinate molecule, Sin304, which comes from the crystallization buffer, was added to the model with the occupancy 0.5. However, a significant amount of positive electron density was observed around that molecule suggesting that we should increase the occupancy of Sin304 to 1.0. When these changes were made, the new QM/MM refinement leads to a better difference density distribution in the binding pocket (Fig. 10b), and ZDD around the ligand 675 decreases (improves) from 2.2 to 1.3 units, and the GBVI/WSA score for the ligand 675 decreases from − 7.11 to − 6.40 kcal/mol. This shift leads to a significant improvement in correlation for the uPA set (the R^2^ moves from 0.61 to 0.74). Furthermore, the residual of the 4FU9 data point improved from − 0.75 to − 0.15.

#### CDK2 structures

As shown in Fig. 5, the structures 4EK5 and 4FKO yield binding affinity predictions (GBVI/WSA score) which deviate significantly from the prediction versus experiment trendline for both conventional refinement and QM/MM refinement and yield residuals of 0.69 and − 0.95 respectively. Upon further review of the QM/MM X-ray refined structure 4EK5, the alternative 'A' conformations of the side chains of the residues Leu32, Lys33, and Lys89 (which the reader will recall are kept by default during the structure preparation step) are not in the agreement with the electron density. Instead, the alternative 'B' conformations in the deposited 4EK5 structure show a better agreement with density. Also, two water molecules (Wat581 and Wat631) show no electron density peaks to justify their placement. A similar situation is observed in the QM/MM refined 4FKO in which the 'B' conformations of the side chains of the residues Val29, Leu78, Lys89, and Met91 were fit to the density, and Wat599 does not have a supporting density peak. These changes were subsequently made to the input structures, and new Phenix/DivCon X-ray refinements were performed leading to significant changes in the final structures. In particular, in the structure 4EK5, the amid group of the ligand 03 K becomes more coplanar with the phenyl group to which it is connected (the corresponding torsion angle is − 11.1° in the new-QM/MM X-ray refined structure as compared to its value of − 16.5° in the original-QM/MM refined structure). Such a rotation might be attributed to the removal of Wat631 in the vicinity of the amid group which may have reduced a steric barrier. In both structures, the ZDD of each ligand decreases (improves) in the new-QM/MM structure while the strain energy remains relatively unchanged. Changes in the GBVI/WSA score were in the range 0.2–0.5 while the overall correlation R^2^ for the CDK2 set increased from 0.60 to 0.71. The residuals 4EK5 and 4FKO also improve to 0.49 and − 0.54 respectively.

### Impact of protomer/tautomer selection: XModeScore results

The XModeScore method [44,45] incorporates both a statistical analysis of the difference density distribution and the local ligand strain energy in order to correctly determine "flip" states and protomer/tautomer states of ligands. As a final step in the process, each of the QM/MM refined structures in the previous step (with any manual modifications noted) were submitted to XModeScore analysis in order to determine proper tautomer/protomer states. The final XModeScore results for all CSAR structures are given in Supplementary Table S3. The results for several CSAR sets presented below demonstrate how the correct choice of ligand tautomer/protomer impacts the predictability of the GBVI/WSA score function. See Figs. 5, 6, 7, and 8 (lines/dots/equations/correlations shown in Blue correspond to XModeScore results).

#### CDK2 structures

**Fig. 11:**
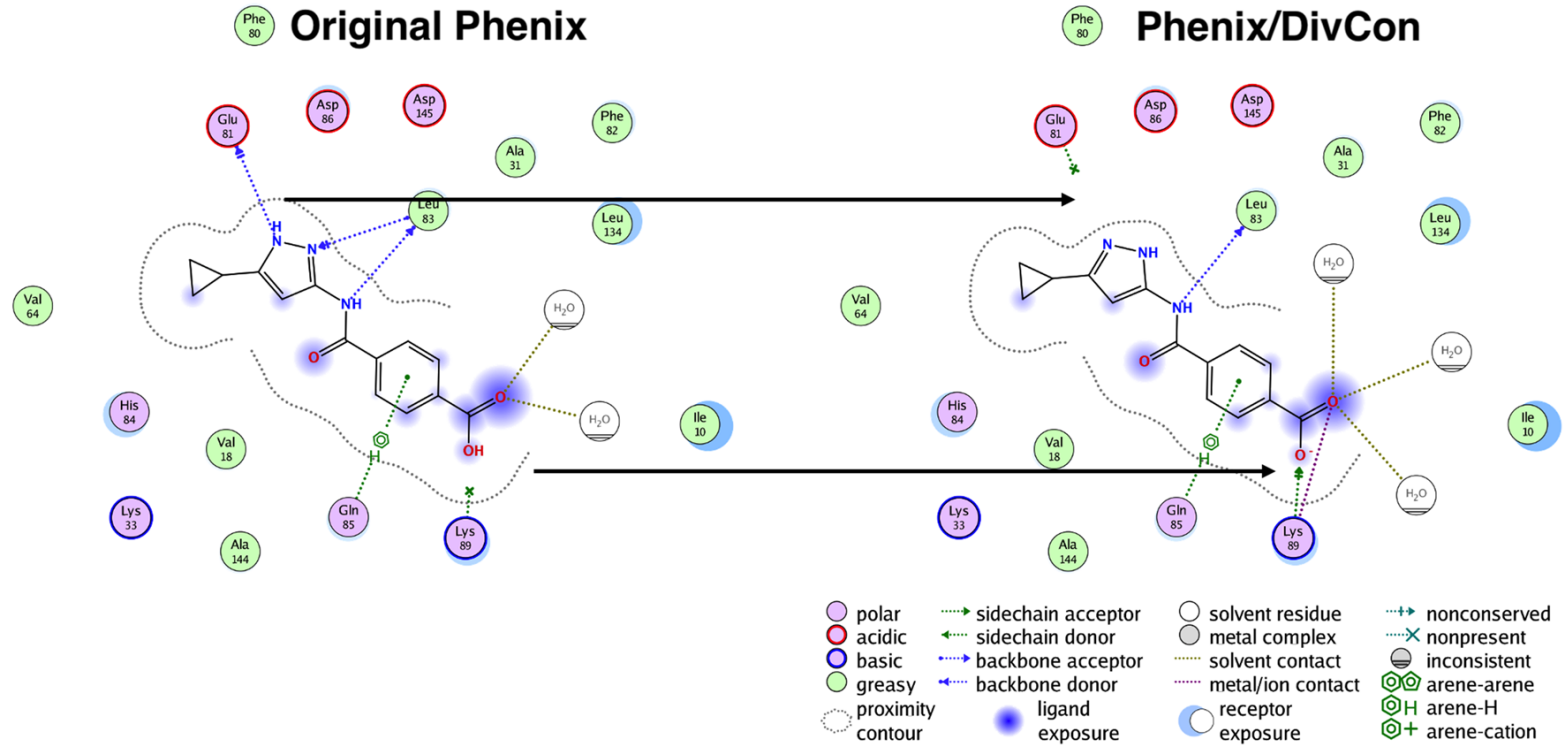
Ligand Interaction diagram for the ligand ID 4CK in the PDB structure 4FKG after the QM/MM and conventional PHENIX refinements. Arrows added to underscore significant structural and interaction changes

As indicated in Table 2, the default protonation states for ligands in 11 out or 15 CDK2 structures prevail as the best tautomeric forms as determined by XModeScore. However, for four structures (4FKP, 4FKQ, 4FKW and 4FKG), XModeScore results show that the best tautomer is different from the one in the initially protonated structures. For example, the best tautomer of the ligand LS5 (PDB 4FKP) is different by the deprotonation of the nitrogen atom of the amino(imino)methylamino group that change GBVI/WSA score by − 0.1 kcal/mol. Similar magnitudes of the change for the scoring function are observed for 4FKQ and 4FKW (Tables 1, 2). The largest structural changes are observed for the ligand 4CK in 4FKG. The default protonation resulted in the protonated carboxyl group –COOH; however, the preferred state of 4CK as determined by XModeScore has a negatively charged carboxyl group which leads to an XModeScore of 1.94 while the default state is assigned a worse score of − 1.08. The tautomer with the unprotonated carboxyl shifts towards Lys89 during the new-QM/MM X-ray refinement, and with some changes in the side chain conformation of Lys89, a stronger H-bond is formed with a OAC^4CK^-NZ^Lys89^ distance of 2.82 Å (versus 3.08 Å in the original-QM/MM refined version with the default tautomer). The interaction diagram for the ligand 4CK (Fig. 11) graphically depicts the strong H-bond mentioned above as well as a more ordered water structure around the tautomer with the unprotonated carboxyl group. Furthermore, the ZDD score is slightly better for the "winning" tautomer, and the calculated binding affinity (GBVI/WSA score) increased from − 6.76 kcal/mol in the original-QM/MM refined structure to − 7.07 kcal/mol in the new-QM/MM refined structure. Taking into account the new GBVI/WSA score values for 4 structures mentioned above the CDK2 set exhibits slightly better correlation (R^2^ = 0.73) versus the previously noted R^2^ = 0.71 with the default protonation. It should be noted that the manually manipulated structures were also included in this analysis (Fig. 5: *Blue* dots/lines).

**Table 2:** Final strain energy, ZDD and GBVI/WSA score values for the best tautomers as determined by XModeScore that are different from the default protonation states after QM/MM and conventional PHENIX refinements

PDB ID	Ligand	Phenix/DivCon (QM/MM)			PHENIX		
		Strain Energy	ZDD	GBVI/WSA	Strain Energy	ZDD	GBVI/WSA
4FKG	4CK	6.53	1.70	− 7.07	21.60	1.97	− 6.86
4FKQ	42K	8.16	4.43	− 8.18	27.36	3.31	− 8.45
4FKW	62K	13.23	4.74	− 9.01	26.51	5.75	− 9.05
4FKP	LS5	10.29	2.73	− 7.66	28.21	14.92	− 6.67
4FU8	2UP	6.65	0.78	− 5.29	14.22	3.01	− 5.49
4FUC	239	9.23	1.97	− 7.54	15.18	2.24	− 7.50
4FSR	HKC	7.01	0.44	− 7.77	18.01	0.84	− 7.64
4FTC	H6K	4.03	2.17	− 7.09	20.71	6.30	− 6.86
4FV7	E94	10.9	2.21	− 9.47	46.56	3.61	− 10.43

#### uPA structures

**Fig. 12:**
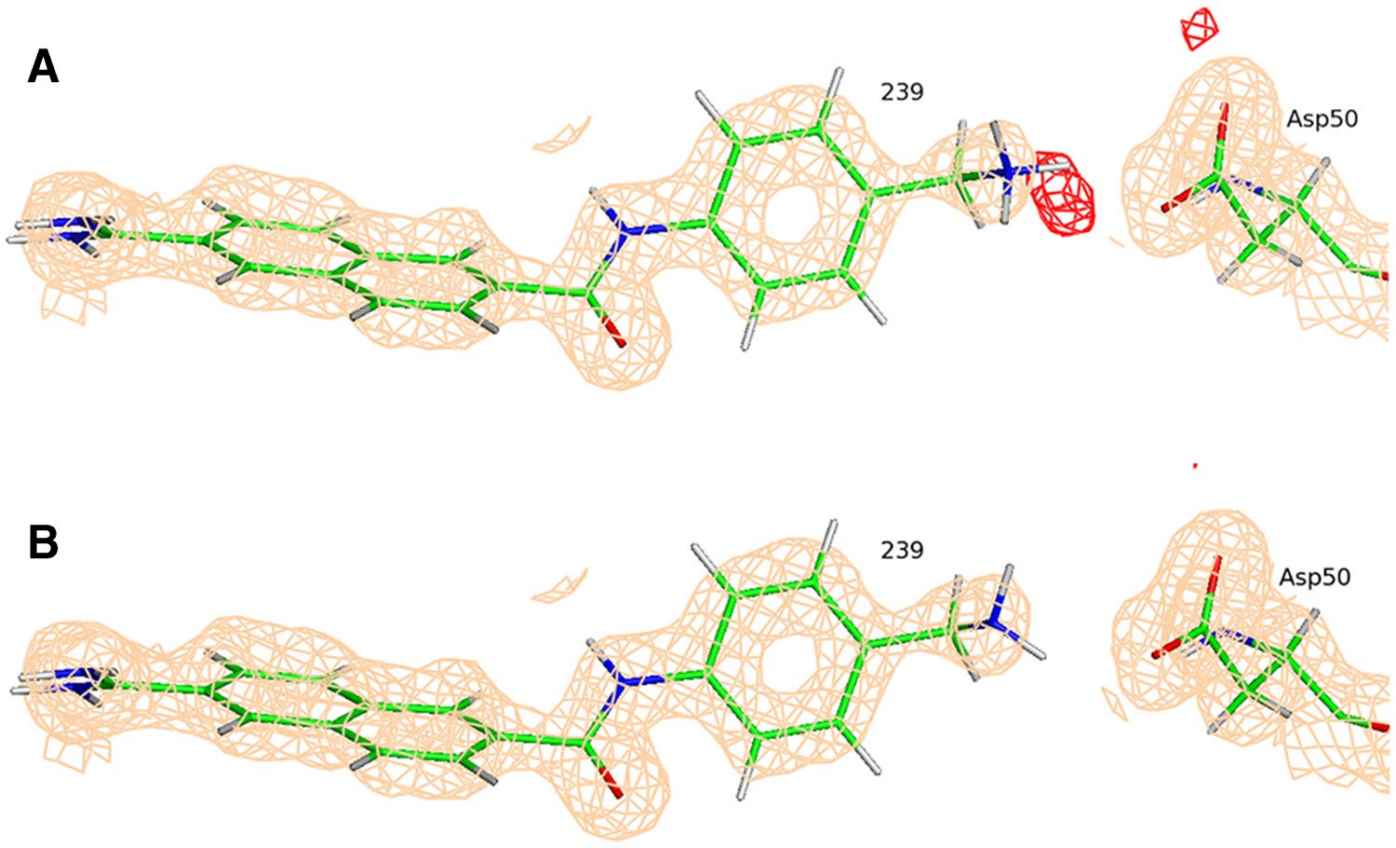
The σ_A_-weighted *mFo-DFc* difference electron density map peaks drawn at 3σ level around the ligand (ligand ID 239) in the PDB structure 4FUC refined with QM/MM for the default (**a**) and XModeScore best tautomers (**b**). The σ_A_-weighted *2mFo-DFc* electron density map is contoured at 1 σ

As was discussed above we changed the tautomeric state of the ligand 675 in the structure 4FU9 based upon the manual examination of the electron density map alone (Fig. 10). XModeScore calculations confirm that this tautomer—with the fully protonated amino(imino)methyl group—is the most favorable one (Fig. 10b) having an XModeScore of 1.28 while the default ligand state depicted on Fig. 10a has the score of − 2.43. Furthermore, according to XModeScore results, the best tautomer of the ligand 2UP in the structure 4FU8 also represents the state with the fully protonated the amino(imino)methyl group. The Phenix/DivCon refinement using this ligand protonation led to the change of GBVI/WSA score from − 5.52 to − 5.29 kcal/mol (Tables 1, 2). Finally, in the structure 4FUC we discovered that the default protonation state of the ligand 239 with the charged ${\text{NH}}_{3}^{+}$ group has a worse XModeScore score than that of the uncharged state having the ${\text{NH}}_{2}$ group. While the latter ligand state weakens the H-bond interaction between the ammonia group and Asp50 (the distance N38^239^-OD2^Asp50^ equals to 2.95 Å in the new-QM/MM structure versus 2.79 Å in the original-QM/MM structure), the new-QM/MM refinement shows that it exhibits better agreement with the experimental density as evidenced by a smaller ZDD value (1.97 units) compared to its magnitude in the original-QM/MM structure (4.42 units) (Fig. 12). Furthermore, a large residual negative density peak seen only in the structure with the ${\text{NH}}_{3}^{+}$ group protonation supports that conclusion. Overall, the correlation R^2^ coefficient for the uPA set shifted significantly from 0.74 to 0.81 when the updated GBVI/WSA score values for these two structures are substituted for the original values in the correlation analysis (Fig. 7: *Blue* dots/lines).

#### CHK1 structures

Finally, for the CHK1 set, two structures (4FTC and 4FSR) were found to have alternative tautomer states which yield better XModeScore's versus than the original states (Table 2, Fig. 8). Specifically, for ligand H6K of PDB 4FTC, the hydrogen atom should be placed on the other nitrogen of pyrazole ring. Both the ZDD value and strain energy associated with the new tautomer is lower (Tables 1, 2) and the GBVI/WSA score became more negative by 0.2 kcal/mol. It is notable that the original protonation state of H6K matches the deposited CIF for this ligand. Overall, using the tautomers determined by XModeScore for 4FTC and 4FSR led to a slightly improved correlation for the CHK1 set (the R^2^ went from 0.75 to 0.77).

### Impact of scoring function choice on binding affinity results

For simplicity and brevity, all of the analyses in the present work focused on the impact of X-ray refinement using the default/recommended score function in MOE: GBVI/WSA. Tables 3 and 4 are also included in order to provide the analogous results using the alternative score functions available in MOE and to compare the various functions available in the platform. We generally observe that GBVI/WSA provides the most consistent performance in two ways: GBVI/WSA is more likely to yield significant correlations for each case, and it appears to be more sensitive to the impact of improved structure. There are some cases in which the London dG (uPA) and Alpha HB (CDK2 and ERK2) score functions also perform well. However, often these functions perform similarly regardless of what manipulations are performed suggesting that—if our goal is to have a strong computational chemistry ↔ structural biology (X-ray crystallography) "feedback loop"—these other scores may be less useful.

**Table 3:** The overall correlation R^2^ for four CSAR target sets calculated based on 5 score functions GBVI/WSA, LDG, ASE, ADG, and AHB

	Phenix/DivCon (QM/MM)					PHENIX				
	GBVI	LDG	ASE	ADG	AHB	GBVI	LDG	ASE	ADG	AHB
CDK2	**0.60(140)**	0.46(*35*)	0.25(*92*)	0.31(*29*)	0.64(*10*)	0.25	0.34	0.13	0.24	**0.58**
ERK2	**0.76(65)**	0.53(47)	0.41(*17*)	0.56(*33*)	0.75(*27*)	0.46	0.36	0.35	0.42	**0.59**
uPA	0.60(*2*)	**0.73**(11)	0.35(*13*)	0.21(***50***)	0.59(*11*)	0.59	**0.66**	0.31	0.14	0.53
CHK1	**0.75**(*15*)	0.48(17)	0.34(*10*)	0.33(*6*)	0.40(***21***)	**0.65**	0.41	0.31	0.31	0.33

The percent change in correlation (R^2^) after QM/MM refinement as compared with conventional PHENIX refinement is given in parenthesis

Highest values in correlation and percent improvement are highlighted in bold

**Table 4:** The overall correlation R^2^ for four CSAR target sets calculated based on 5 score functions GBVI/WSA, LDG, ASE, ADG, and AHB for the hand modified (^a^) and XModeScore (^b^) chosen structures

	GBV	LDG	ASE	ADG	AHB
CDK2	**0.71 (184)**^**a**^**, 0.73(192)**^**b**^	0.48 (*41*), 0.52(*53*)	0.28 (*115*), 0.27 (*108*)	0.35 (*46*), 0.47 (*96*)	0.67 (*16*), 0.67 (*16*)
ERK2	**0.81 (76)**^**b**^	0.53 (*47*)	0.42 (*20*)	0.57(*36*)	0.76 (*29*)
uPA	0.74 (*25*)^a^,** 0.81 (37)**^**b**^	**0.78 (18),** 0.80 (*21*)	0.38 (*23*), 0.41 (*32*)	0.24 (*71*), 0.28(*100*)	0.60 (*13*), 0.61 (*15*)
CHK1	**0.77 (18)**^**b**^	0.49 (*20*)	0.34 (*10*)	0.34 (*10*)	0.42 (*27*)

The percent change in correlation (R^2^) after QM/MM refinement as compared to the conventional PHENIX refinement is given in parenthesis

Highest values in correlation and percent improvement are highlighted in bold

## Conclusions

There are many structural metrics used to evaluate the quality of protein structures and hence the performance of a given crystallographic method. These metrics include overall R factors (R_free_ and R_work_), MolProbity statistics [59], local ligand strain energy as well as more sophisticated computed Z-score of the difference density or ZDD [44]. Using these metrics, we have demonstrated that Phenix/DivCon (QM/MM) X-ray refinement [35, 36] yields superior quality protein:ligand complex structures as compared to conventional PHENIX refinement when challenged with the X-ray models available in popular and well curated Community Structure Activity Resource (CSAR) benchmark set. Furthermore, we have shown that XModeScore [44, 45]—which couples model strain with model experimental density agreement—can be used to successfully determine the correct tautomer/protomer states of the ligands (and active sites) of interest. In this work we also showed that when provided with more accurate QM/MM refined X-ray models, we can use conventional score functions (such as the GBVI/WSA score function found in MOE) to "flag" X-ray models for further crystallographic consideration. Specifically, we used the correlation between the experimentally determined binding affinities of the ligands available in the CSAR protein set and the predicted GBVI/WSA scores calculated based on the refined structures as an additional metric to indicate those cases which provide opportunities for further X-ray density-driven manipulation. Upon subsequent QM/MM refinement, these new X-ray structures give rise to better predicted versus experiment correlation coefficients suggesting that not only were these structures more accurate (as measured by the aforementioned crystallographic metrics), but they were more chemically descriptive of the key protein:ligand interactions important to the SBDD effort. Through this protocol, we have shown that score function predictability, and likely by extension overall SBDD performance, can be greatly enhanced by choosing the correct conformations of the receptor side chains, positions of water molecules as well as the correct protonation/tautomeric state of the ligand. With the proper, QM/MM based refinement tools, this synergistic approach can be replicated within industrial and academic pharmaceutical laboratories.

Going forward, we will continue the development of the Phenix/DivCon (and BUSTER/DivCon) method through the addition of two key improvements. First, the QM method used exclusively in the present work was the PM6 Hamiltonian as originally published [48, 49] and subsequently implemented by QuantumBio staff in the DivCon Discovery Suite. We will explore the impact of the PM6-D3H4 hydrogen bonding and dispersion correction approach added to PM6 by Řezáč and Hobza [67, 68]. Second, since the initial ligand positions were not resampled and all X-ray refinement was performed on the original ligand poses (unless otherwise indicated), the refinements as presented were limited to the same radius of convergence of the published structure. Therefore, only the local ligand strain energy or LLSE is reported in order to better gauge the impact of the change of functional alone. In future work, in order to mitigate the docking (placement) induced strain and to more accurately measure the global ligand strain, this approach will be coupled with the MovableType Conformational Search (MT_CS_) and Docking (MT_Dock_) fast free energy methods recently implemented in QuantumBio's software [69–71].

## Supplementary information

All resulting PDB and MTZ files are provided in the following file: https://downloads.quantumbioinc.com/media/tutorials/MT/csar_paper.tar.gz.

## Electronic supplementary material

Below is the link to the electronic supplementary material.

Supplementary file1 (DOCX 103 kb)
