# Supplementary material for: The critical role of QM/MM X-ray refinement and accurate tautomer/protomer determination in structure-based drug design
Source: J Comput Aided Mol Des. 2020 Oct 27;35(4):433–51. doi: 10.1007/s10822-020-00354-6 (PMC8018927; doi:10.1007/s10822-020-00354-6)
Supplement: Supplementary file 1 — Supplementary file1 (DOCX 103 kb) [file 10822_2020_354_MOESM1_ESM.docx]

**SUPPORTING INFORMATION**

**The critical role of QM/MM X-ray refinement and accurate tautomer/protomer determination in Structure Based Drug Design**

Oleg Borbulevych^a^, Roger I. Martin^a^, and Lance M. Westerhoff^*,a^

^a^QuantumBio Inc, 2790 West College Ave, Suite 900, State College, PA, 16801, USA

**Supplementary** **Table 1.** Ligand Strain energies and ZDD values, as well as Molprobity statistics for the original 55 CSAR PDB structures (see Publication Table 1 for corresponding PHENIX and Phenix/DivCon refinement results)

| PDB ID | Res | Ligand ID | Strain Energy | ZDD | GBVI/  WSA | Clash  Score | MolProbity Score |
| --- | --- | --- | --- | --- | --- | --- | --- |
| **CDK2** |  |  |  |  |  |  |  |
| 4FKL | 1.26 | CK2 | 16.06 | 9.86 | -5.73 | 2.1 | 1.03 |
| 4EK8 | 1.7 | 16K | 9.33 | 15.18 | -7.05 | 2.61 | 1.05 |
| 3SW4 | 1.7 | 18K | 16.38 | 5.80 | -7.32 | 1.74 | 1 |
| 3SW7 | 1.8 | 19K | 19.09 | 6.59 | -8.03 | 2.43 | 1.02 |
| 4EK4 | 1.26 | 1CK | 24.63 | 8.08 | -6.43 | 1.51 | 1.04 |
| 4FKO | 1.55 | 20K | 17.69 | 6.74 | -7.99 | 1.07 | 0.9 |
| 4FKP | 1.6 | LS5 | 70.05 | 2.87 | -8.30 | 2.21 | 1 |
| 4FKQ | 1.75 | 42K | 91.03 | 3.31 | -8.44 | 1.74 | 0.93 |
| 4FKR | 1.9 | 45K | 51.61 | 3.38 | -8.04 | 1.53 | 0.9 |
| 4FKS | 1.54 | 46K | 22.85 | 11.61 | -8.76 | 1.37 | 1.07 |
| 4FKU | 1.47 | 60K | 31.36 | 4.96 | -8.41 | 1.66 | 0.92 |
| 4FKW | 1.8 | 62K | 57.73 | 5.24 | -8.09 | 1.99 | 0.97 |
| 4EK5 | 1.6 | 03K | 48.63 | 4.12 | -7.10 | 1.9 | 0.95 |
| 4FKG | 1.51 | 4CK | 56.59 | 2.81 | -6.92 | 2.17 | 0.99 |
| 4FKI | 1.6 | 09K | 24.2 | 3.43 | -7.53 | 3.05 | 1.1 |
| **CHK1** |  |  |  |  |  |  |  |
| 4FSQ | 2.4 | HK3 | 30.77 | 5.35 | -6.24 | 1.21 | 1.07 |
| 4FSR | 2.49 | HKC | 31.83 | 3.53 | -7.30 | 1.43 | 1.06 |
| 4FST | 1.9 | HK4 | 11.62 | 3.84 | -8.68 | 2.2 | 1.15 |
| 4FSU | 2.09 | HK5 | 13.98 | 4.53 | -8.12 | 1.95 | 1.1 |
| 4FT5 | 2.39 | H2K | 27.56 | 4.63 | -7.63 | 2.15 | 1.12 |
| 4FT3 | 2.5 | H1K | 23.55 | 3.26 | -5.40 | 1.15 | 1.01 |
| 4FSN | 2.1 | A58 | 32.38 | 6.07 | -7.80 | 1.86 | 1.01 |
| 4FSZ | 2.3 | HK8 | 14.84 | 2.74 | -6.25 | 2.09 | 1.24 |
| 4FT7 | 2.2 | H3K | 28.88 | 7.21 | -8.61 | 1.85 | 1.07 |
| 4FSM | 2.2 | HK1 | 12.18 | 4.40 | -7.18 | 3.09 | 1.36 |
| 4FSY | 2.29 | HK7 | 10.37 | 2.49 | -6.54 | 1.4 | 0.94 |
| 4FT0 | 2.3 | HK9 | 26.76 | 3.32 | -8.29 | 1.91 | 1.18 |
| 4FT9 | 2.2 | H4K | 17.54 | 2.92 | -7.29 | 1.39 | 1 |
| 4FTA | 2.4 | H5K | 18.78 | 2.71 | -5.43 | 2.84 | 1.3 |
| 4FSW | 2.3 | HK6 | 11.78 | 6.93 | -5.37 | 1.9 | 1.14 |
| 4FTC | 2 | H6K | 18.66 | 3.17 | -6.07 | 1.65 | 1.05 |
|  |  |  |  |  |  |  |  |
|  |  |  |  |  |  |  |  |
| **ERK2** |  |  |  |  |  |  |  |
| 4FUX | 2.2 | E75 | 28.2 | 7.86 | -6.87 | 2.15 | 1.16 |
| 4FUY | 2 | EK2 | 43.79 | 7.31 | -5.09 | 3.13 | 1.26 |
| 4FV0 | 2.09 | EK3 | 28.75 | 7.32 | -5.59 | 2.48 | 1.21 |
| 4FV1 | 1.99 | EK4 | 29.59 | 6.79 | -8.02 | 1.06 | 0.98 |
| 4FV2 | 2 | EK5 | 60.1 | 2.16 | -7.99 | 1.41 | 1.21 |
| 4FV3 | 2.2 | EK6 | 33.31 | 10.21 | -6.93 | 1.93 | 1.16 |
| 4FV4 | 2.5 | EK7 | 30.38 | 4.34 | -7.72 | 0.57 | 0.95 |
| 4FV5 | 2.4 | EK9 | 28.78 | 2.37 | -9.04 | 0.92 | 0.92 |
| 4FV6 | 2.5 | E57 | 38.06 | 2.40 | -10.41 | 0.74 | 0.94 |
| 4FV7 | 1.9 | E94 | 46.48 | 6.34 | -10.19 | 1.76 | 1.02 |
| 4FV8 | 2 | E63 | 42.29 | 2.99 | -8.28 | 2.19 | 1.15 |
| 4FV9 | 2.11 | E71 | 20.82 | 3.93 | -8.14 | 0.37 | 0.78 |
| **syk** |  |  |  |  |  |  |  |
| 4YJQ | 1.34 | 4DK | 34.74 | 5.56 | -9.47 | 1.37 | 1.1 |
| 4YJR | 1.32 | 4DJ | 31.61 | 2.94 | -8.69 | 0.91 | 1.01 |
| 4YJT | 1.52 | 4DQ | 26.78 | 4.66 | -8.82 | 1.13 | 1 |
| 4YJU | 1.67 | 4DO | 30.12 | 3.22 | -8.84 | 1.38 | 1.06 |
| 4YJV | 1.65 | 4DT | 67.26 | 5.07 | -9.16 | 1.6 | 1.14 |
| **uPA** |  |  |  |  |  |  |  |
| 4FUD | 2 | 6UP | 16.4 | 3.60 | -5.20 | 2.55 | 1.4 |
| 4FUE | 2 | 7UP | 17.73 | 5.15 | -7.02 | 2.03 | 1.13 |
| 4FU7 | 2 | 1UP | 14.99 | 3.01 | -6.73 | 2.59 | 1.42 |
| 4FU8 | 2.2 | 2UP | 15.91 | 4.50 | -5.45 | 1.81 | 1.22 |
| 4FU9 | 1.6 | 675 | 10.63 | 4.13 | -7.42 | 1.01 | 0.89 |
| 4FUB | 1.9 | 4UP | 45.98 | 2.56 | -6.63 | 5.1 | 1.28 |
| 4FUC | 1.72 | 239 | 21.66 | 5.35 | -7.66 | 0.75 | 0.83 |

**Supplementary** **Table 2.** R_work_ and R_free_ values after ONIOM and conventional PHENIX refinements of 55 CSAR PDB structures.

| PDBID | Resolution | Rwork/Rfree | Rwork/Rfree |
| --- | --- | --- | --- |
| 4FKL | 1.26 | 0.1877/0.2126 | 0.1841/0.2111 |
| 4EK8 | 1.7 | 0.1890/0.2262 | 0.1869/0.2253 |
| 3SW4 | 1.7 | 0.1858/0.2168 | 0.1841/0.2166 |
| 3SW7 | 1.8 | 0.1879/0.2145 | 0.1857/0.2127 |
| 4EK4 | 1.26 | 0.1955/0.2117 | 0.1891/0.2089 |
| 4FKO | 1.55 | 0.1856/0.2018 | 0.1818/0.1998 |
| 4FKP | 1.6 | 0.1974/0.2118 | 0.1917/0.2091 |
| 4FKQ | 1.75 | 0.1743/0.2024 | 0.1709/0.1998 |
| 4FKR | 1.9 | 0.1780/0.2107 | 0.1769/0.2145 |
| 4FKS | 1.54 | 0.1904/0.2123 | 0.1880/0.2146 |
| 4FKU | 1.47 | 0.1792/0.2037 | 0.1759/0.2021 |
| 4FKW | 1.8 | 0.1813/0.2151 | 0.1793/0.2138 |
| 4EK5 | 1.6 | 0.1816/0.2133 | 0.1778/0.2126 |
| 4FKG | 1.51 | 0.1902/0.2132 | 0.1877/0.2120 |
| 4FKI | 1.6 | 0.1894/0.2162 | 0.1872/0.2160 |
| 4FSQ | 2.4 | 0.1666/0.1878 | 0.1660/0.1884 |
| 4FSR | 2.49 | 0.1638/0.1993 | 0.1629/0.1965 |
| 4FST | 1.9 | 0.1672/0.1939 | 0.1649/0.1909 |
| 4FSU | 2.09 | 0.1661/0.1890 | 0.1641/0.1887 |
| 4FT5 | 2.39 | 0.1693/0.2233 | 0.1682/0.2230 |
| 4FT3 | 2.5 | 0.1558/0.1881 | 0.1523/0.1795 |
| 4FSN | 2.1 | 0.1685/0.2133 | 0.1661/0.2120 |
| 4FSZ | 2.3 | 0.1598/0.2196 | 0.1589/0.2164 |
| 4FT7 | 2.2 | 0.1609/0.1937 | 0.1585/0.1909 |
| 4FSM | 2.2 | 0.1706/0.2104 | 0.1685/0.2056 |
| 4FSY | 2.29 | 0.1638/0.1906 | 0.1627/0.1896 |
| 4FT0 | 2.3 | 0.1653/0.1933 | 0.1640/0.1928 |
| 4FT9 | 2.2 | 0.1553/0.1862 | 0.1537/0.1843 |
| 4FTA | 2.4 | 0.1600/0.1795 | 0.1590/0.1764 |
| 4FSW | 2.3 | 0.1662/0.2009 | 0.1659/0.2017 |
| 4FTC | 2 | 0.1664/0.1990 | 0.1637/0.1922 |
| 4FUX | 2.2 | 0.1754/0.2053 | 0.1733/0.2034 |
| 4FUY | 2 | 0.1775/0.2133 | 0.1753/0.2135 |
| 4FV0 | 2.09 | 0.1673/0.2171 | 0.1650/0.2196 |
| 4FV1 | 1.99 | 0.1689/0.1937 | 0.1659/0.1907 |
| 4FV2 | 2 | 0.1753/0.1964 | 0.1738/0.1974 |
| 4FV3 | 2.2 | 0.1749/0.2125 | 0.1725/0.2079 |
| 4FV4 | 2.5 | 0.1947/0.2323 | 0.1930/0.2335 |
| 4FV5 | 2.4 | 0.1746/0.2483 | 0.1725/0.2446 |
| 4FV6 | 2.5 | 0.1901/0.2557 | 0.1889/0.2582 |
| 4FV7 | 1.9 | 0.1721/0.2014 | 0.1695/0.2015 |
| 4FV8 | 2 | 0.1785/0.2322 | 0.1759/0.2324 |
| 4FV9 | 2.11 | 0.1892/0.2095 | 0.1869/0.2075 |
| 4YJQ | 1.34 | 0.1754/0.1909 | 0.1705/0.1906 |
| 4YJR | 1.32 | 0.1708/0.1860 | 0.1663/0.1836 |
| 4YJT | 1.52 | 0.1726/0.2009 | 0.1684/0.1979 |
| 4YJU | 1.67 | 0.1781/0.2132 | 0.1755/0.2142 |
| 4YJV | 1.65 | 0.1705/0.2126 | 0.1662/0.2091 |
| 4FUD | 2 | 0.1623/0.1993 | 0.1553/0.1945 |
| 4FUE | 2 | 0.1507/0.1874 | 0.1473/0.1855 |
| 4FU7 | 2 | 0.1690/0.2282 | 0.1677/0.2278 |
| 4FU8 | 2.2 | 0.1591/0.2049 | 0.1568/0.2036 |
| 4FU9 | 1.6 | 0.1503/0.1808 | 0.1461/0.1817 |
| 4FUB | 1.9 | 0.1449/0.1829 | 0.1423/0.1846 |
| 4FUC | 1.72 | 0.1472/0.1723 | 0.1439/0.1692 |

**Supplementary** **Table 3.** XModeScore results based on the ONIOM refinement for 55 structures of the CSAR set.

|  |  | **CDK2 Set** |  |  |  |
| --- | --- | --- | --- | --- | --- |
|  |  | 4FKL |  |  |  |
| Species | Residue | StrainEnergy | RSCC | ZDD | XModeScore |
| 0_0_0_0_0 | /A/CK2/300// | 2.575 | 0.96 | 4.657 | 2 |
| 1_0_0_0_1 | /A/CK2/300// | 22.316 | 0.961 | 4.697 | -2 |
|  |  |  |  |  |  |
|  |  | 4EK8 |  |  |  |
| Species | Residue | StrainEnergy | RSCC | ZDD | XModeScore |
| 0_0_0_0_0 | /A/16K/301// | 8.166 | 0.917 | 11.316 | 2 |
| 1_0_0_0_1 | /A/16K/301// | 17.886 | 0.916 | 12.187 | -2 |
|  |  |  |  |  |  |
|  |  | 3SW4 |  |  |  |
| Species | Residue | StrainEnergy | RSCC | ZDD | XModeScore |
| 1_0_0_0_0 | /A/18K/299// | 5.59 | 0.946 | 3.24 | 2.079 |
| 0_0_0_0_-1 | /A/18K/299// | 6.367 | 0.947 | 3.573 | 0.89 |
| 2_1_0_0_0 | /A/18K/299// | 6.134 | 0.945 | 3.872 | -0.108 |
| 3_0_0_0_1 | /A/18K/299// | 35.397 | 0.942 | 4.005 | -2.86 |
|  |  |  |  |  |  |
|  |  | 3SW7 |  |  |  |
| Species | Residue | StrainEnergy | RSCC | ZDD | XModeScore |
| 0_0_0_0_0 | /A/19K/299// | 7.165 | 0.96 | 3.304 | 2 |
| 1_0_0_0_1 | /A/19K/299// | 25.129 | 0.958 | 3.812 | -2 |
|  |  |  |  |  |  |
|  |  | 4EK4 |  |  |  |
| Species | Residue | StrainEnergy | RSCC | ZDD | XModeScore |
| 3_0_0_0_0 | /A/1CK/301// | 3.803 | 0.958 | 6.889 | 1.497 |
| 0_0_0_0_-1 | /A/1CK/301// | 3.293 | 0.954 | 7.515 | 1.148 |
| 1_1_0_0_-1 | /A/1CK/301// | 4.49 | 0.956 | 7.378 | 0.177 |
| 4_1_0_0_0 | /A/1CK/301// | 3.265 | 0.95 | 8.276 | 0.152 |
| 2_1_0_1_-1 | /A/1CK/301// | 3.207 | 0.945 | 9.219 | -1.06 |
| 6_0_0_0_1 | /A/1CK/301// | 6.124 | 0.954 | 7.762 | -1.914 |
|  |  |  |  |  |  |
|  |  |  |  |  |  |
|  |  | 4FKO |  |  |  |
| Species | Residue | StrainEnergy | RSCC | ZDD | XModeScore |
| 0_0_0_0_0 | /A/20K/301// | 6.532 | 0.965 | 2.936 | 2 |
| 1_0_0_0_1 | /A/20K/301// | 14.765 | 0.964 | 2.941 | -2 |
|  |  |  |  |  |  |
|  |  | 4FKQ |  |  |  |
| Species | Residue | Strain | Energy | RSCC | ZDD |
| 46_1_1_4_1 | /A/42K/301// | 7.619 | 0.968 | 6.343 | 2.824 |
| 65_2_1_3_1 | /A/42K/301// | 7.858 | 0.969 | 6.403 | 2.735 |
| 50_1_2_3_1 | /A/42K/301// | 13.364 | 0.969 | 6.335 | 2.143 |
| 1_1_0_0_-1 | /A/42K/301// | 10.136 | 0.966 | 6.961 | 1.902 |
| 68_2_1_6_1 | /A/42K/301// | 8.432 | 0.965 | 7.556 | 1.509 |
| 15_1_1_0_0 | /A/42K/301// | 13.481 | 0.965 | 7.418 | 1.043 |
| 51_1_2_4_1 | /A/42K/301// | 11.526 | 0.964 | 7.984 | 0.709 |
| 7_1_0_6_-1 | /A/42K/301// | 15.656 | 0.963 | 7.912 | 0.287 |
| 49_1_2_2_1 | /A/42K/301// | 23.258 | 0.964 | 7.318 | -0.028 |
| 48_1_2_1_1 | /A/42K/301// | 15.689 | 0.962 | 8.24 | -0.047 |
| 20_1_1_5_0 | /A/42K/301// | 18.504 | 0.962 | 8.41 | -0.554 |
| 2_1_0_1_-1 | /A/42K/301// | 15.537 | 0.96 | 8.97 | -0.761 |
| 81_2_2_9_1 | /A/42K/301// | 16.667 | 0.96 | 8.868 | -0.793 |
| 75_2_2_3_1 | /A/42K/301// | 26.311 | 0.963 | 7.957 | -1.035 |
| 78_2_2_6_1 | /A/42K/301// | 22.96 | 0.959 | 8.874 | -1.554 |
| 47_1_2_0_1 | /A/42K/301// | 19.946 | 0.958 | 9.31 | -1.63 |
| 80_2_2_8_1 | /A/42K/301// | 17.459 | 0.955 | 10.03 | -2.054 |
| 36_0_0_0_1 | /A/42K/301// | 39.59 | 0.964 | 7.634 | -2.303 |
| 41_1_0_4_1 | /A/42K/301// | 34.282 | 0.961 | 8.357 | -2.393 |
|  |  |  |  |  |  |
|  |  |  |  |  |  |
|  |  |  |  |  |  |
|  |  | 4FKP |  |  |  |
| Species | Residue | StrainEnergy | RSCC | ZDD | XModeScore |
| 1_1_0_0_-1 | /A/LS5/301// | 10.287 | 0.961 | 4.169 | 2 |
| 6_1_2_1_-1 | /A/LS5/301// | 11.998 | 0.96 | 3.99 | 1.804 |
| 3_1_1_0_-1 | /A/LS5/301// | 13.55 | 0.96 | 3.632 | 1.764 |
| 0_0_0_0_-1 | /A/LS5/301// | 12.37 | 0.958 | 4.095 | 1.662 |
| 12_2_3_0_-1 | /A/LS5/301// | 10.805 | 0.961 | 4.585 | 1.613 |
| 10_2_1_0_-1 | /A/LS5/301// | 13.721 | 0.961 | 3.83 | 1.594 |
| 15_0_0_0_0 | /A/LS5/301// | 17.226 | 0.956 | 5.454 | -0.194 |
| 19_1_3_0_0 | /A/LS5/301// | 15.944 | 0.956 | 5.978 | -0.318 |
| 17_1_1_0_0 | /A/LS5/301// | 18.864 | 0.951 | 7.006 | -1.581 |
| 18_1_2_0_0 | /A/LS5/301// | 23.335 | 0.951 | 6.64 | -2.162 |
| 16_1_0_0_0 | /A/LS5/301// | 22.54 | 0.948 | 7.152 | -2.37 |
| 21_0_0_0_1 | /A/LS5/301// | 27.846 | 0.948 | 7.797 | -3.812 |
|  |  |  |  |  |  |
|  |  |  |  |  |  |
|  |  | 4FKR |  |  |  |
| Species | Residue | StrainEnergy | RSCC | ZDD | XModeScore |
| 4_0_0_0_0 | /A/45K/301// | 10.813 | 0.971 | 3.997 | 1.706 |
| 1_1_0_0_-1 | /A/45K/301// | 10.958 | 0.969 | 4.305 | 1.501 |
| 7_1_1_0_0 | /A/45K/301// | 11.196 | 0.969 | 4.434 | 1.397 |
| 2_1_0_1_-1 | /A/45K/301// | 12.203 | 0.969 | 4.557 | 1.216 |
| 0_0_0_0_-1 | /A/45K/301// | 10.681 | 0.968 | 4.838 | 1.201 |
| 10_0_0_0_1 | /A/45K/301// | 19.984 | 0.967 | 5.066 | 0.09 |
| 11_1_0_0_1 | /A/45K/301// | 12.141 | 0.959 | 7.71 | -0.725 |
| 13_1_2_0_1 | /A/45K/301// | 29.676 | 0.958 | 7.755 | -2.581 |
| 3_1_0_2_-1 | /A/45K/301// | 38.559 | 0.955 | 8.238 | -3.806 |
|  |  |  |  |  |  |
|  |  | 4FKS |  |  |  |
| Species | Residue | StrainEnergy | RSCC | ZDD | XModeScore |
| 10_0_0_0_0 | /A/46K/301// | 16.4 | 0.967 | 3.387 | 2.078 |
| 6_1_1_2_-1 | /A/46K/301// | 13.8 | 0.968 | 3.644 | 2.023 |
| 4_1_1_0_-1 | /A/46K/301// | 17.115 | 0.965 | 3.888 | 1.044 |
| 11_1_0_0_0 | /A/46K/301// | 14.795 | 0.965 | 4.224 | 0.8 |
| 13_1_1_0_0 | /A/46K/301// | 15.18 | 0.966 | 4.209 | 0.766 |
| 21_1_0_0_1 | /A/46K/301// | 19.094 | 0.966 | 3.941 | 0.63 |
| 0_0_0_0_-1 | /A/46K/301// | 14.527 | 0.964 | 4.567 | 0.214 |
| 3_1_0_2_-1 | /A/46K/301// | 14.639 | 0.964 | 4.692 | -0.035 |
| 1_1_0_0_-1 | /A/46K/301// | 13.77 | 0.964 | 4.846 | -0.178 |
| 23_1_2_0_1 | /A/46K/301// | 19.293 | 0.965 | 4.618 | -0.645 |
| 22_1_1_0_1 | /A/46K/301// | 19.554 | 0.963 | 4.663 | -0.77 |
| 9_2_0_2_-1 | /A/46K/301// | 37.423 | 0.965 | 4.165 | -2.721 |
| 20_0_0_0_1 | /A/46K/301// | 24.765 | 0.965 | 5.536 | -3.206 |
|  |  |  |  |  |  |
|  |  |  |  |  |  |
|  |  | 4FKU |  |  |  |
| Species | Residue | StrainEnergy | RSCC | ZDD | XModeScore |
| 20_0_0_0_0 | /A/60K/303// | 32.06 | 0.975 | 5.96 | 2.583 |
| 0_0_0_0_-1 | /A/60K/303// | 31.024 | 0.975 | 6.213 | 2.022 |
| 13_2_1_0_-1 | /A/60K/303// | 51.211 | 0.976 | 5.852 | 1.557 |
| 7_1_2_0_-1 | /A/60K/303// | 37.49 | 0.975 | 6.241 | 1.516 |
| 5_1_1_1_-1 | /A/60K/303// | 45.236 | 0.976 | 6.107 | 1.327 |
| 4_1_1_0_-1 | /A/60K/303// | 47.436 | 0.975 | 6.05 | 1.32 |
| 16_2_2_0_-1 | /A/60K/303// | 41.752 | 0.973 | 6.363 | 0.924 |
| 1_1_0_0_-1 | /A/60K/303// | 40.518 | 0.974 | 6.412 | 0.884 |
| 25_1_2_0_0 | /A/60K/303// | 53.935 | 0.976 | 6.152 | 0.626 |
| 2_1_0_1_-1 | /A/60K/303// | 43.606 | 0.973 | 6.622 | 0.151 |
| 10_2_0_0_-1 | /A/60K/303// | 50.487 | 0.973 | 6.537 | -0.101 |
| 23_1_1_0_0 | /A/60K/303// | 47.815 | 0.973 | 6.7 | -0.326 |
| 38_1_2_0_1 | /A/60K/303// | 45.693 | 0.972 | 6.963 | -0.84 |
| 35_0_0_0_1 | /A/60K/303// | 66.011 | 0.975 | 6.487 | -1.026 |
| 39_1_3_0_1 | /A/60K/303// | 54.481 | 0.973 | 6.805 | -1.04 |
| 37_1_1_0_1 | /A/60K/303// | 55.942 | 0.971 | 7.318 | -2.417 |
| 40_1_4_0_1 | /A/60K/303// | 85.585 | 0.972 | 6.839 | -3.228 |
| 36_1_0_0_1 | /A/60K/303// | 85.555 | 0.973 | 7.122 | -3.931 |
|  |  |  |  |  |  |
|  |  | 4FKW |  |  |  |
| Species | Residue | StrainEnergy | RSCC | ZDD | XModeScore |
| 4_1_1_0_-1 | /A/62K/301// | 13.209 | 0.95 | 8.757 | 2.455 |
| 15_1_2_0_0 | /A/62K/301// | 14.273 | 0.95 | 8.906 | 1.738 |
| 7_2_0_0_-1 | /A/62K/301// | 18.102 | 0.952 | 8.335 | 1.185 |
| 10_0_0_0_0 | /A/62K/301// | 14.883 | 0.949 | 9.151 | 1.036 |
| 24_1_3_0_1 | /A/62K/301// | 17.455 | 0.95 | 8.63 | 0.917 |
| 2_1_0_1_-1 | /A/62K/301// | 17.679 | 0.95 | 9.001 | 0.144 |
| 5_1_1_1_-1 | /A/62K/301// | 13.962 | 0.946 | 10.06 | -0.243 |
| 0_0_0_0_-1 | /A/62K/301// | 18.342 | 0.948 | 9.36 | -0.79 |
| 6_1_1_2_-1 | /A/62K/301// | 18.747 | 0.945 | 9.723 | -1.624 |
| 20_0_0_0_1 | /A/62K/301// | 19.296 | 0.946 | 9.996 | -2.352 |
| 21_1_0_0_1 | /A/62K/301// | 20.963 | 0.947 | 9.679 | -2.467 |
|  |  |  |  |  |  |
|  |  |  |  |  |  |
|  |  | 4EK5 |  |  |  |
| Species | Residue | StrainEnergy | RSCC | ZDD | XModeScore |
| 3_0_0_0_0 | /A/03K/300// | 4.795 | 0.953 | 5.833 | 1.876 |
| 0_0_0_0_-1 | /A/03K/300// | 5.447 | 0.955 | 5.673 | 1.659 |
| 1_1_0_0_-1 | /A/03K/300// | 4.768 | 0.95 | 6.414 | 1.176 |
| 4_1_0_0_0 | /A/03K/300// | 5.076 | 0.949 | 6.718 | 0.603 |
| 6_0_0_0_1 | /A/03K/300// | 8.504 | 0.948 | 7.488 | -2.54 |
| 2_1_0_1_-1 | /A/03K/300// | 8.094 | 0.944 | 7.891 | -2.775 |
|  |  |  |  |  |  |
|  |  | 4FKG |  |  |  |
| Species | Residue | StrainEnergy | RSCC | ZDD | XModeScore |
| 6_1_1_2_-1 | /A/4CK/300// | 4.242 | 0.942 | 5.204 | 1.939 |
| 8_2_0_1_-1 | /A/4CK/300// | 4.548 | 0.943 | 5.128 | 1.855 |
| 5_1_1_1_-1 | /A/4CK/300// | 7.294 | 0.948 | 4.159 | 1.472 |
| 4_1_1_0_-1 | /A/4CK/300// | 7.466 | 0.947 | 4.125 | 1.415 |
| 11_1_0_0_0 | /A/4CK/300// | 4.057 | 0.939 | 5.822 | 1.263 |
| 9_2_0_2_-1 | /A/4CK/300// | 6.641 | 0.943 | 5.08 | 0.685 |
| 19_2_2_0_0 | /A/4CK/300// | 4.921 | 0.938 | 6.008 | 0.517 |
| 0_0_0_0_-1 | /A/4CK/300// | 6.672 | 0.942 | 5.348 | 0.327 |
| 16_1_2_1_0 | /A/4CK/300// | 7.511 | 0.942 | 5.32 | -0.131 |
| 10_0_0_0_0 | /A/4CK/300// | 6.639 | 0.935 | 6.474 | -1.085 |
| 21_1_0_0_1 | /A/4CK/300// | 7.68 | 0.937 | 6.388 | -1.588 |
| 2_1_0_1_-1 | /A/4CK/300// | 7.359 | 0.936 | 6.601 | -1.67 |
| 22_1_1_0_1 | /A/4CK/300// | 9.749 | 0.939 | 5.88 | -2.16 |
| 1_1_0_0_-1 | /A/4CK/300// | 9.411 | 0.936 | 6.572 | -2.84 |
|  |  |  |  |  |  |
|  |  | 4FKI |  |  |  |
| Species | Residue | StrainEnergy | RSCC | ZDD | XModeScore |
| 3_0_0_0_0 | /A/09K/301// | 4.927 | 0.949 | 7.122 | 2.26 |
| 0_0_0_0_-1 | /A/09K/301// | 5.167 | 0.948 | 7.192 | 2.066 |
| 2_1_0_1_-1 | /A/09K/301// | 5.003 | 0.946 | 7.767 | 1.249 |
| 4_1_0_0_0 | /A/09K/301// | 5.221 | 0.944 | 8.258 | 0.423 |
| 6_0_0_0_1 | /A/09K/301// | 9.488 | 0.942 | 8.692 | -1.781 |
| 5_1_1_0_0 | /A/09K/301// | 12.185 | 0.944 | 8.169 | -1.958 |
| 1_1_0_0_-1 | /A/09K/301// | 9.58 | 0.939 | 8.984 | -2.259 |
|  |  | **CHK1 Set** |  |  |  |
|  |  | 4FSQ |  |  |  |
| Species | Residue | StrainEnergy | RSCC | ZDD | XModeScore |
| 0_0_0_0_0 | /A/HK3/300// | 13.687 | 0.956 | 1.929 | 1.981 |
| 1_0_0_0_1 | /A/HK3/300// | 15.223 | 0.956 | 1.882 | 0.101 |
| 2_1_0_0_1 | /A/HK3/300// | 15.18 | 0.955 | 2.273 | -2.082 |
|  |  |  |  |  |  |
|  |  | 4FSR |  |  |  |
| Species | Residue | StrainEnergy | RSCC | ZDD | XModeScore |
| 7_0_0_0_1 | /A/HKC/300// | 8.149 | 0.968 | 2.057 | 1.56 |
| 3_1_0_1_0 | /A/HKC/300// | 8.962 | 0.964 | 2.121 | 1.429 |
| 15_1_1_3_1 | /A/HKC/300// | 8.671 | 0.968 | 2.18 | 1.42 |
| 2_1_0_0_0 | /A/HKC/300// | 6.779 | 0.967 | 2.87 | 1.152 |
| 1_0_0_0_0 | /A/HKC/300// | 7.67 | 0.965 | 3.271 | 0.781 |
| 4_1_0_2_0 | /A/HKC/300// | 8.396 | 0.961 | 3.284 | 0.693 |
| 0_0_0_0_-1 | /A/HKC/300// | 11.529 | 0.963 | 3.708 | 0.063 |
| 5_1_0_3_0 | /A/HKC/300// | 7.307 | 0.962 | 5.787 | -0.903 |
| 10_1_0_2_1 | /A/HKC/300// | 8.386 | 0.962 | 5.668 | -0.938 |
| 18_2_0_2_1 | /A/HKC/300// | 8.708 | 0.963 | 5.744 | -1.025 |
| 14_1_1_2_1 | /A/HKC/300// | 9.253 | 0.961 | 5.892 | -1.186 |
| 6_1_0_4_0 | /A/HKC/300// | 41.653 | 0.965 | 3.478 | -3.045 |
|  |  |  |  |  |  |
|  |  | 4FST |  |  |  |
| Species | Residue | StrainEnergy | RSCC | ZDD | XModeScore |
| 1_0_0_0_0 | /A/HK4/301// | 9.972 | 0.965 | 1.885 | 1.726 |
| 0_0_0_0_-1 | /A/HK4/301// | 12.483 | 0.965 | 1.8 | 0.928 |
| 3_0_0_0_1 | /A/HK4/301// | 15.388 | 0.961 | 2.37 | -2.654 |
|  |  |  |  |  |  |
|  |  | 4FT5 |  |  |  |
| Species | Residue | StrainEnergy | RSCC | ZDD | XModeScore |
| 5_0_0_0_0 | /A/H2K/300// | 12.394 | 0.909 | 8.872 | 2.117 |
| 0_0_0_0_-1 | /A/H2K/300// | 11.928 | 0.911 | 8.964 | 1.81 |
| 2_1_0_1_-1 | /A/H2K/300// | 16.776 | 0.906 | 9.213 | 0.376 |
| 15_0_0_0_1 | /A/H2K/300// | 19.087 | 0.908 | 9.171 | 0.307 |
| 17_1_0_1_1 | /A/H2K/300// | 23.808 | 0.911 | 9.181 | -0.202 |
| 22_2_0_0_1 | /A/H2K/300// | 29.084 | 0.91 | 9.212 | -0.842 |
| 16_1_0_0_1 | /A/H2K/300// | 44.336 | 0.914 | 8.823 | -0.867 |
| 24_2_2_0_1 | /A/H2K/300// | 28.423 | 0.905 | 9.713 | -2.699 |
|  |  |  |  |  |  |
|  |  | 4FT3 |  |  |  |
| Species | Residue | StrainEnergy | RSCC | ZDD | XModeScore |
| 4_0_0_0_0 | /A/H1K/301// | 11.246 | 0.967 | 1.42 | 2.131 |
| 0_0_0_0_-1 | /A/H1K/301// | 6.642 | 0.958 | 2.41 | 1.176 |
| 1_1_0_0_-1 | /A/H1K/301// | 9.605 | 0.959 | 2.244 | 1.079 |
| 12_1_1_0_1 | /A/H1K/301// | 15.454 | 0.965 | 1.839 | 1.006 |
| 7_1_1_0_0 | /A/H1K/301// | 15.764 | 0.964 | 1.951 | 0.8 |
| 2_1_0_1_-1 | /A/H1K/301// | 6.346 | 0.956 | 3.329 | -0.176 |
| 10_0_0_0_1 | /A/H1K/301// | 33.424 | 0.958 | 2.41 | -1.959 |
| 11_1_0_0_1 | /A/H1K/301// | 19.824 | 0.955 | 3.467 | -1.962 |
| 13_1_2_0_1 | /A/H1K/301// | 25.711 | 0.954 | 3.098 | -2.094 |
|  |  |  |  |  |  |
|  |  | 4FSN |  |  |  |
| Species | Residue | StrainEnergy | RSCC | ZDD | XModeScore |
| 5_0_0_0_0 | /A/A58/301// | 14.243 | 0.929 | 4.078 | 1.992 |
| 20_1_2_0_1 | /A/A58/301// | 17.462 | 0.931 | 3.963 | 1.172 |
| 12_2_0_0_0 | /A/A58/301// | 14.974 | 0.932 | 4.23 | 1.043 |
| 15_0_0_0_1 | /A/A58/301// | 17.925 | 0.932 | 3.992 | 0.86 |
| 1_1_0_0_-1 | /A/A58/301// | 15.651 | 0.931 | 4.258 | 0.645 |
| 23_2_1_0_1 | /A/A58/301// | 20.287 | 0.932 | 3.819 | 0.641 |
| 6_1_0_0_0 | /A/A58/301// | 14.885 | 0.932 | 4.429 | 0.225 |
| 24_2_2_0_1 | /A/A58/301// | 19.83 | 0.929 | 3.982 | 0.126 |
| 0_0_0_0_-1 | /A/A58/301// | 15.103 | 0.931 | 4.445 | 0.069 |
| 3_1_0_2_-1 | /A/A58/301// | 14.595 | 0.93 | 4.502 | 0.032 |
| 9_1_1_0_0 | /A/A58/301// | 15.98 | 0.93 | 4.376 | 0.005 |
| 19_1_1_1_1 | /A/A58/301// | 14.961 | 0.929 | 4.559 | -0.363 |
| 10_1_1_1_0 | /A/A58/301// | 20.342 | 0.93 | 4.082 | -0.512 |
| 21_1_2_1_1 | /A/A58/301// | 15.684 | 0.928 | 4.564 | -0.68 |
| 16_1_0_0_1 | /A/A58/301// | 21.843 | 0.93 | 4.008 | -0.806 |
| 17_1_0_1_1 | /A/A58/301// | 19.498 | 0.929 | 4.234 | -0.821 |
| 18_1_1_0_1 | /A/A58/301// | 17.835 | 0.93 | 4.461 | -1.116 |
| 2_1_0_1_-1 | /A/A58/301// | 20.78 | 0.93 | 4.507 | -2.513 |
|  |  |  |  |  |  |
|  |  | 4FSZ |  |  |  |
| Species | Residue | StrainEnergy | RSCC | ZDD | XModeScore |
| 10_0_0_0_0 | /A/HK8/301// | 7.239 | 0.955 | 1.81 | 1.581 |
| 17_2_0_0_0 | /A/HK8/301// | 9.137 | 0.957 | 1.72 | 0.857 |
| 14_1_1_1_0 | /A/HK8/301// | 8.444 | 0.955 | 1.83 | 0.811 |
| 11_1_0_0_0 | /A/HK8/301// | 7.977 | 0.954 | 1.93 | 0.674 |
| 8_2_0_1_-1 | /A/HK8/301// | 6.539 | 0.949 | 2.176 | 0.502 |
| 9_2_0_2_-1 | /A/HK8/301// | 7.354 | 0.949 | 2.117 | 0.273 |
| 2_1_0_1_-1 | /A/HK8/301// | 11.125 | 0.955 | 1.663 | -0.055 |
| 0_0_0_0_-1 | /A/HK8/301// | 11.95 | 0.96 | 1.572 | -0.161 |
| 1_1_0_0_-1 | /A/HK8/301// | 11.179 | 0.954 | 1.733 | -0.371 |
| 3_1_0_2_-1 | /A/HK8/301// | 12.243 | 0.958 | 1.582 | -0.371 |
| 4_1_1_0_-1 | /A/HK8/301// | 8.917 | 0.948 | 2.056 | -0.378 |
| 21_1_0_0_1 | /A/HK8/301// | 8.18 | 0.953 | 2.206 | -0.562 |
| 22_1_1_0_1 | /A/HK8/301// | 9.84 | 0.953 | 2.03 | -0.803 |
| 5_1_1_1_-1 | /A/HK8/301// | 9.242 | 0.947 | 2.409 | -1.998 |
|  |  |  |  |  |  |
|  |  | 4FT7 |  |  |  |
| Species | Residue | StrainEnergy | RSCC | ZDD | XModeScore |
| 4_0_0_0_0 | /A/H3K/301// | 12.577 | 0.964 | 4.28 | 2.513 |
| 1_1_0_0_-1 | /A/H3K/301// | 13.352 | 0.962 | 4.376 | 2.14 |
| 0_0_0_0_-1 | /A/H3K/301// | 18.26 | 0.962 | 4.615 | 0.852 |
| 7_1_1_0_0 | /A/H3K/301// | 28.946 | 0.962 | 4.194 | 0.78 |
| 13_1_2_0_1 | /A/H3K/301// | 24.454 | 0.961 | 4.651 | -0.004 |
| 12_1_1_0_1 | /A/H3K/301// | 28.253 | 0.959 | 4.675 | -0.534 |
| 2_1_0_1_-1 | /A/H3K/301// | 29.281 | 0.96 | 4.802 | -1.028 |
| 11_1_0_0_1 | /A/H3K/301// | 24.957 | 0.957 | 5.419 | -2.298 |
| 10_0_0_0_1 | /A/H3K/301// | 40.355 | 0.961 | 4.82 | -2.421 |
|  |  |  |  |  |  |
|  |  | 4FSM |  |  |  |
| Species | Residue | Strain | Energy | RSCC | ZDD |
| 1_0_0_0_0 | /A/HK1/301// | 5.384 | 0.962 | 2.005 | 1.759 |
| 0_0_0_0_-1 | /A/HK1/301// | 7.079 | 0.961 | 2.02 | 1.026 |
| 3_0_0_0_1 | /A/HK1/301// | 12.179 | 0.956 | 2.226 | -2.785 |
|  |  |  |  |  |  |
|  |  | 4FSY |  |  |  |
| Species | Residue | Strain | Energy | RSCC | ZDD |
| 3_0_0_0_0 | /A/HK7/301// | 8.993 | 0.962 | 1.9 | 2.036 |
| 4_1_0_0_0 | /A/HK7/301// | 10.523 | 0.961 | 1.925 | 1.301 |
| 2_1_0_1_-1 | /A/HK7/301// | 9.029 | 0.954 | 2.233 | 1.139 |
| 1_1_0_0_-1 | /A/HK7/301// | 12.462 | 0.954 | 2.117 | -0.056 |
| 5_1_1_0_0 | /A/HK7/301// | 8.842 | 0.951 | 2.941 | -0.656 |
| 6_0_0_0_1 | /A/HK7/301// | 12.337 | 0.956 | 2.799 | -1.81 |
| 0_0_0_0_-1 | /A/HK7/301// | 15.459 | 0.96 | 2.338 | -1.953 |
|  |  |  |  |  |  |
|  |  | 4FT0 |  |  |  |
| Species | Residue | Strain | Energy | RSCC | ZDD |
| 10_0_0_0_0 | /A/HK9/301// | 10.783 | 0.964 | 1.233 | 2.408 |
| 9_2_0_2_-1 | /A/HK9/301// | 9.236 | 0.962 | 1.5 | 2.196 |
| 2_1_0_1_-1 | /A/HK9/301// | 12.598 | 0.963 | 1.353 | 1.149 |
| 1_1_0_0_-1 | /A/HK9/301// | 11.007 | 0.961 | 1.701 | 0.676 |
| 11_1_0_0_0 | /A/HK9/301// | 16.365 | 0.966 | 0.999 | 0.637 |
| 19_2_2_0_0 | /A/HK9/301// | 14.759 | 0.964 | 1.26 | 0.473 |
| 5_1_1_1_-1 | /A/HK9/301// | 16.551 | 0.965 | 1.126 | 0.108 |
| 16_1_2_1_0 | /A/HK9/301// | 14.217 | 0.962 | 1.524 | -0.198 |
| 21_1_0_0_1 | /A/HK9/301// | 14.959 | 0.962 | 1.457 | -0.309 |
| 22_1_1_0_1 | /A/HK9/301// | 14.563 | 0.96 | 1.534 | -0.394 |
| 8_2_0_1_-1 | /A/HK9/301// | 15.105 | 0.964 | 1.519 | -0.592 |
| 4_1_1_0_-1 | /A/HK9/301// | 15.386 | 0.963 | 1.656 | -1.199 |
| 6_1_1_2_-1 | /A/HK9/301// | 15.179 | 0.959 | 2.044 | -2.456 |
| 0_0_0_0_-1 | /A/HK9/301// | 15.624 | 0.961 | 1.997 | -2.499 |
|  |  |  |  |  |  |
|  |  | 4FT9 |  |  |  |
| Species | Residue | StrainEnergy | RSCC | ZDD | XModeScore |
| 5_0_0_0_0 | /A/H4K/300// | 5.257 | 0.962 | 2.567 | 2.171 |
| 12_2_0_0_0 | /A/H4K/300// | 5.627 | 0.962 | 2.582 | 2.111 |
| 6_1_0_0_0 | /A/H4K/300// | 5.233 | 0.96 | 2.915 | 1.682 |
| 2_1_0_1_-1 | /A/H4K/300// | 6.027 | 0.96 | 2.93 | 1.576 |
| 0_0_0_0_-1 | /A/H4K/300// | 6.335 | 0.961 | 3.015 | 1.424 |
| 1_1_0_0_-1 | /A/H4K/300// | 8.571 | 0.96 | 2.945 | 1.285 |
| 19_1_1_1_1 | /A/H4K/300// | 6.566 | 0.959 | 3.124 | 1.244 |
| 15_0_0_0_1 | /A/H4K/300// | 7.481 | 0.959 | 3.224 | 1.007 |
| 17_1_0_1_1 | /A/H4K/300// | 10.217 | 0.958 | 3.428 | 0.428 |
| 10_1_1_1_0 | /A/H4K/300// | 21.183 | 0.96 | 2.836 | 0.106 |
| 3_1_0_2_-1 | /A/H4K/300// | 22.161 | 0.961 | 2.957 | -0.168 |
| 18_1_1_0_1 | /A/H4K/300// | 18.829 | 0.957 | 3.526 | -0.621 |
| 16_1_0_0_1 | /A/H4K/300// | 29.028 | 0.958 | 3.389 | -1.507 |
| 22_2_0_0_1 | /A/H4K/300// | 17.843 | 0.952 | 4.553 | -1.971 |
| 23_2_1_0_1 | /A/H4K/300// | 20.807 | 0.95 | 4.665 | -2.443 |
| 24_2_2_0_1 | /A/H4K/300// | 23.584 | 0.948 | 4.95 | -3.139 |
| 20_1_2_0_1 | /A/H4K/300// | 36.865 | 0.954 | 3.988 | -3.183 |
|  |  |  |  |  |  |
|  |  | 4FTA |  |  |  |
| Species | Residue | StrainEnergy | RSCC | ZDD | XModeScore |
| 15_0_0_0_0 | /A/H5K/301// | 5.894 | 0.952 | 2.353 | 2.596 |
| 16_1_0_0_0 | /A/H5K/301// | 6.855 | 0.95 | 2.475 | 2.32 |
| 5_1_1_0_-1 | /A/H5K/301// | 6.881 | 0.949 | 2.684 | 2.001 |
| 12_2_0_3_-1 | /A/H5K/301// | 9.139 | 0.951 | 2.688 | 1.778 |
| 6_1_1_1_-1 | /A/H5K/301// | 6.873 | 0.947 | 2.854 | 1.745 |
| 2_1_0_1_-1 | /A/H5K/301// | 9.382 | 0.949 | 2.738 | 1.679 |
| 1_1_0_0_-1 | /A/H5K/301// | 6.289 | 0.946 | 3.074 | 1.469 |
| 39_1_1_1_1 | /A/H5K/301// | 10.467 | 0.947 | 2.955 | 1.247 |
| 10_2_0_1_-1 | /A/H5K/301// | 8.351 | 0.947 | 3.105 | 1.224 |
| 0_0_0_0_-1 | /A/H5K/301// | 6.903 | 0.943 | 3.587 | 0.635 |
| 37_1_0_1_1 | /A/H5K/301// | 11.879 | 0.941 | 3.63 | 0.092 |
| 23_1_2_1_0 | /A/H5K/301// | 24.387 | 0.946 | 2.932 | -0.056 |
| 47_2_3_0_1 | /A/H5K/301// | 20.356 | 0.945 | 3.37 | -0.33 |
| 45_2_1_0_1 | /A/H5K/301// | 22.117 | 0.946 | 3.381 | -0.517 |
| 48_2_4_0_1 | /A/H5K/301// | 28.814 | 0.944 | 3.322 | -1.07 |
| 31_2_2_0_0 | /A/H5K/301// | 27.86 | 0.943 | 3.462 | -1.191 |
| 7_1_1_2_-1 | /A/H5K/301// | 24.877 | 0.942 | 3.905 | -1.574 |
| 38_1_1_0_1 | /A/H5K/301// | 35.236 | 0.939 | 3.942 | -2.625 |
| 44_2_0_0_1 | /A/H5K/301// | 23.788 | 0.936 | 4.831 | -2.866 |
| 36_1_0_0_1 | /A/H5K/301// | 34.707 | 0.937 | 4.372 | -3.223 |
| 49_2_5_0_1 | /A/H5K/301// | 33.095 | 0.935 | 4.547 | -3.333 |
|  |  |  |  |  |  |
|  |  | 4FSW |  |  |  |
| Species | Residue | StrainEnergy | RSCC | ZDD | XModeScore |
| 3_0_0_0_0 | /A/HK6/301// | 6.776 | 0.957 | 2.671 | 1.012 |
| 6_0_0_0_1 | /A/HK6/301// | 7.363 | 0.958 | 2.58 | 0.779 |
| 1_1_0_0_-1 | /A/HK6/301// | 6.113 | 0.953 | 3.135 | 0.35 |
| 4_1_0_0_0 | /A/HK6/301// | 6.34 | 0.952 | 3.325 | -0.32 |
| 2_1_0_1_-1 | /A/HK6/301// | 5.221 | 0.945 | 3.7 | -0.391 |
| 0_0_0_0_-1 | /A/HK6/301// | 9.228 | 0.957 | 2.859 | -1.43 |
|  |  |  |  |  |  |
|  |  | 4FTC |  |  |  |
| Species | Residue | StrainEnergy | RSCC | ZDD | XModeScore |
| 16_1_0_0_0 | /A/H6K/301// | 6.078 | 0.948 | 3.771 | 2.34 |
| 15_0_0_0_0 | /A/H6K/301// | 4.834 | 0.944 | 4.23 | 1.909 |
| 2_1_0_1_-1 | /A/H6K/301// | 5.189 | 0.944 | 4.274 | 1.83 |
| 0_0_0_0_-1 | /A/H6K/301// | 4.56 | 0.943 | 4.342 | 1.802 |
| 1_1_0_0_-1 | /A/H6K/301// | 6.799 | 0.944 | 4.362 | 1.594 |
| 5_1_1_0_-1 | /A/H6K/301// | 5.428 | 0.942 | 4.489 | 1.56 |
| 39_1_1_1_1 | /A/H6K/301// | 9.893 | 0.942 | 4.595 | 1.069 |
| 6_1_1_1_-1 | /A/H6K/301// | 4.988 | 0.937 | 5.086 | 0.903 |
| 37_1_0_1_1 | /A/H6K/301// | 11.914 | 0.941 | 4.873 | 0.58 |
| 45_2_1_0_1 | /A/H6K/301// | 19.676 | 0.939 | 4.863 | -0.047 |
| 35_0_0_0_1 | /A/H6K/301// | 10.04 | 0.934 | 5.735 | -0.267 |
| 38_1_1_0_1 | /A/H6K/301// | 34.977 | 0.941 | 4.945 | -1.403 |
| 47_2_3_0_1 | /A/H6K/301// | 21.348 | 0.934 | 6.018 | -1.527 |
| 44_2_0_0_1 | /A/H6K/301// | 30.96 | 0.936 | 5.492 | -1.707 |
| 36_1_0_0_1 | /A/H6K/301// | 39.614 | 0.938 | 5.28 | -2.174 |
| 48_2_4_0_1 | /A/H6K/301// | 30.928 | 0.931 | 6.41 | -2.771 |
| 49_2_5_0_1 | /A/H6K/301// | 31.003 | 0.927 | 7.197 | -3.692 |
|  |  |  |  |  |  |
|  |  | **ERK2 Set** |  |  |  |
|  |  | 4FUX |  |  |  |
| Species | Residue | StrainEnergy | RSCC | ZDD | XModeScore |
| 0_0_0_0_0 | /A/E75/401// | 8.819 | 0.915 | 5.155 | 2 |
| 1_0_0_0_1 | /A/E75/401// | 9.83 | 0.917 | 4.879 | -2 |
|  |  |  |  |  |  |
|  |  | 4FUY |  |  |  |
| Species | Residue | StrainEnergy | RSCC | ZDD | XModeScore |
| 3_0_0_0_0 | /A/EK2/401// | 10.396 | 0.956 | 3.101 | 1.956 |
| 4_1_0_0_0 | /A/EK2/401// | 6.373 | 0.956 | 3.521 | 0.941 |
| 6_0_0_0_1 | /A/EK2/401// | 6.047 | 0.956 | 3.672 | 0.516 |
| 2_1_0_1_-1 | /A/EK2/401// | 33.083 | 0.955 | 3.447 | -0.266 |
| 1_1_0_0_-1 | /A/EK2/401// | 33.441 | 0.955 | 3.519 | -0.497 |
| 0_0_0_0_-1 | /A/EK2/401// | 6.069 | 0.953 | 4.291 | -1.301 |
| 5_1_1_0_0 | /A/EK2/401// | 58.369 | 0.954 | 3.356 | -1.349 |
|  |  |  |  |  |  |
|  |  |  |  |  |  |
|  |  | 4FV0 |  |  |  |
| Species | Residue | StrainEnergy | RSCC | ZDD | XModeScore |
| 4_0_0_0_0 | /A/EK3/401// | 11.174 | 0.941 | 1.152 | 2.784 |
| 13_1_2_0_1 | /A/EK3/401// | 13.365 | 0.943 | 1.122 | 2.039 |
| 2_1_0_1_-1 | /A/EK3/401// | 12.142 | 0.941 | 1.35 | 0.716 |
| 10_0_0_0_1 | /A/EK3/401// | 13.55 | 0.94 | 1.274 | 0.706 |
| 7_1_1_0_0 | /A/EK3/401// | 15.55 | 0.942 | 1.253 | -0.031 |
| 5_1_0_0_0 | /A/EK3/401// | 12.907 | 0.939 | 1.461 | -0.546 |
| 12_1_1_0_1 | /A/EK3/401// | 19.253 | 0.942 | 1.192 | -1.202 |
| 11_1_0_0_1 | /A/EK3/401// | 15.965 | 0.938 | 1.381 | -1.267 |
| 0_0_0_0_-1 | /A/EK3/401// | 15.353 | 0.938 | 1.448 | -1.547 |
| 1_1_0_0_-1 | /A/EK3/401// | 15.515 | 0.944 | 1.452 | -1.652 |
|  |  |  |  |  |  |
|  |  | 4FV1 |  |  |  |
| Species | Residue | StrainEnergy | RSCC | ZDD | XModeScore |
| 6_0_0_0_0 | /A/EK4/401// | 38.444 | 0.959 | 2.691 | 1.766 |
| 3_1_1_0_-1 | /A/EK4/401// | 21.836 | 0.958 | 2.838 | 1.041 |
| 1_1_0_0_-1 | /A/EK4/401// | 14.779 | 0.959 | 2.895 | 0.766 |
| 2_1_0_1_-1 | /A/EK4/401// | 39.692 | 0.958 | 2.886 | 0.411 |
| 0_0_0_0_-1 | /A/EK4/401// | 46.896 | 0.96 | 2.906 | 0.155 |
| 10_0_0_0_1 | /A/EK4/401// | 190.91 | 0.954 | 3.185 | -4.139 |
|  |  |  |  |  |  |
|  |  | 4FV2 |  |  |  |
| Species | Residue | StrainEnergy | RSCC | ZDD | XModeScore |
| 1_0_0_0_0 | /A/EK5/401// | 9.086 | 0.958 | 2.265 | 0.668 |
| 5_0_0_0_1 | /A/EK5/401// | 33.089 | 0.958 | 1.603 | 0.479 |
| 7_1_0_1_1 | /A/EK5/401// | 33.897 | 0.959 | 1.733 | 0.016 |
| 0_0_0_0_-1 | /A/EK5/401// | 13.857 | 0.951 | 2.505 | -0.484 |
| 6_1_0_0_1 | /A/EK5/401// | 34.342 | 0.958 | 1.951 | -0.68 |
|  |  |  |  |  |  |
|  |  | 4FV3 |  |  |  |
| Species | Residue | StrainEnergy | RSCC | ZDD | XModeScore |
| 10_0_0_0_0 | /A/EK6/401// | 20.315 | 0.929 | 8.632 | 2.629 |
| 24_1_3_0_1 | /A/EK6/401// | 27.193 | 0.926 | 9.265 | 0.986 |
| 0_0_0_0_-1 | /A/EK6/401// | 28.206 | 0.926 | 9.257 | 0.961 |
| 1_1_0_0_-1 | /A/EK6/401// | 46.682 | 0.925 | 9.443 | -0.206 |
| 7_2_0_0_-1 | /A/EK6/401// | 28.613 | 0.923 | 9.808 | -0.238 |
| 4_1_1_0_-1 | /A/EK6/401// | 57.673 | 0.926 | 9.393 | -0.555 |
| 20_0_0_0_1 | /A/EK6/401// | 99.787 | 0.928 | 9.042 | -1.551 |
| 9_2_0_2_-1 | /A/EK6/401// | 46.127 | 0.919 | 10.303 | -2.026 |
|  |  |  |  |  |  |
|  |  | 4FV4 |  |  |  |
| Species | Residue | StrainEnergy | RSCC | ZDD | XModeScore |
| 1_0_0_0_0 | /A/EK7/401// | 14.212 | 0.927 | 3.143 | 2 |
| 0_0_0_0_-1 | /A/EK7/401// | 14.737 | 0.925 | 3.239 | -2 |
|  |  |  |  |  |  |
|  |  | 4FV5 |  |  |  |
| Species | Residue | StrainEnergy | RSCC | ZDD | XModeScore |
| 4_0_0_0_0 | /A/EK9/401// | 15.204 | 0.966 | 1.592 | 1.735 |
| 5_1_0_0_0 | /A/EK9/401// | 13.979 | 0.964 | 1.605 | 1.732 |
| 0_0_0_0_-1 | /A/EK9/401// | 13.421 | 0.966 | 1.735 | 0.965 |
| 11_1_0_0_1 | /A/EK9/401// | 16.415 | 0.964 | 1.719 | 0.881 |
| 3_1_0_2_-1 | /A/EK9/401// | 12.711 | 0.964 | 1.8 | 0.61 |
| 10_0_0_0_1 | /A/EK9/401// | 23.767 | 0.965 | 1.725 | 0.384 |
| 6_1_0_1_0 | /A/EK9/401// | 14.174 | 0.964 | 1.83 | 0.331 |
| 1_1_0_0_-1 | /A/EK9/401// | 25.01 | 0.961 | 1.942 | -1.032 |
| 2_1_0_1_-1 | /A/EK9/401// | 25.123 | 0.96 | 2.178 | -2.493 |
| 12_1_1_0_1 | /A/EK9/401// | 69.189 | 0.959 | 1.836 | -3.115 |
|  |  |  |  |  |  |
|  |  | 4FV6 |  |  |  |
| Species | Residue | StrainEnergy | RSCC | ZDD | XModeScore |
| 2_0_0_0_0 | /A/E57/401// | 19.271 | 0.97 | 1.406 | 2.487 |
| 1_1_0_0_-1 | /A/E57/401// | 20.344 | 0.968 | 1.853 | -0.682 |
| 0_0_0_0_-1 | /A/E57/401// | 22.268 | 0.969 | 1.77 | -1.805 |
|  |  |  |  |  |  |
|  |  | 4FV7 |  |  |  |
| Species | Residue | StrainEnergy | RSCC | ZDD | XModeScore |
| 1_1_0_0_-1 | /A/E94/401// | 12.387 | 0.953 | 2.844 | 2 |
| 3_0_0_0_0 | /A/E94/401// | 25.423 | 0.954 | 3.155 | -2 |
|  |  |  |  |  |  |
|  |  | 4FV8 |  |  |  |
| Species | Residue | StrainEnergy | RSCC | ZDD | XModeScore |
| 35_0_0_0_0 | /A/E63/401// | 11.576 | 0.955 | 4.362 | 2.426 |
| 9_1_2_2_-1 | /A/E63/401// | 14.188 | 0.957 | 4.141 | 2.318 |
| 24_2_3_2_-1 | /A/E63/401// | 16.074 | 0.955 | 4.306 | 1.64 |
| 43_1_3_1_0 | /A/E63/401// | 11.55 | 0.953 | 4.811 | 1.604 |
| 18_2_1_2_-1 | /A/E63/401// | 13.902 | 0.955 | 4.618 | 1.496 |
| 55_2_9_0_0 | /A/E63/401// | 14.345 | 0.955 | 4.574 | 1.488 |
| 17_2_1_1_-1 | /A/E63/401// | 15.231 | 0.954 | 4.517 | 1.419 |
| 11_1_3_1_-1 | /A/E63/401// | 12.726 | 0.953 | 4.813 | 1.368 |
| 20_2_2_1_-1 | /A/E63/401// | 10.878 | 0.951 | 5.035 | 1.323 |
| 12_1_3_2_-1 | /A/E63/401// | 19.018 | 0.956 | 4.353 | 0.972 |
| 10_1_3_0_-1 | /A/E63/401// | 13.24 | 0.952 | 5.122 | 0.697 |
| 36_1_0_0_0 | /A/E63/401// | 20.719 | 0.955 | 4.358 | 0.627 |
| 30_2_5_2_-1 | /A/E63/401// | 20.001 | 0.955 | 4.465 | 0.571 |
| 21_2_2_2_-1 | /A/E63/401// | 11.434 | 0.95 | 5.467 | 0.417 |
| 26_2_4_1_-1 | /A/E63/401// | 18.533 | 0.954 | 4.733 | 0.366 |
| 0_0_0_0_-1 | /A/E63/401// | 25.421 | 0.957 | 4.046 | 0.271 |
| 27_2_4_2_-1 | /A/E63/401// | 17.394 | 0.953 | 4.967 | 0.161 |
| 45_1_4_1_0 | /A/E63/401// | 16.995 | 0.953 | 5.012 | 0.158 |
| 57_1_0_0_1 | /A/E63/401// | 24.317 | 0.956 | 4.276 | 0.065 |
| 53_2_7_0_0 | /A/E63/401// | 20.692 | 0.954 | 4.724 | -0.044 |
| 8_1_2_1_-1 | /A/E63/401// | 17.047 | 0.951 | 5.165 | -0.135 |
| 1_1_0_0_-1 | /A/E63/401// | 27.311 | 0.956 | 4.267 | -0.511 |
| 58_1_1_0_1 | /A/E63/401// | 24.204 | 0.955 | 4.624 | -0.554 |
| 19_2_2_0_-1 | /A/E63/401// | 17.621 | 0.95 | 5.356 | -0.601 |
| 7_1_2_0_-1 | /A/E63/401// | 17.175 | 0.95 | 5.466 | -0.716 |
| 4_1_1_0_-1 | /A/E63/401// | 25.612 | 0.955 | 4.677 | -0.93 |
| 5_1_1_1_-1 | /A/E63/401// | 26.925 | 0.955 | 4.546 | -0.948 |
| 61_1_4_0_1 | /A/E63/401// | 18.824 | 0.949 | 5.589 | -1.268 |
| 62_1_5_0_1 | /A/E63/401// | 20.782 | 0.95 | 5.553 | -1.59 |
| 56_0_0_0_1 | /A/E63/401// | 24.717 | 0.951 | 5.305 | -1.911 |
| 3_1_0_2_-1 | /A/E63/401// | 27.892 | 0.953 | 5.029 | -2.029 |
| 23_2_3_1_-1 | /A/E63/401// | 20.071 | 0.948 | 6.049 | -2.364 |
| 29_2_5_1_-1 | /A/E63/401// | 20.086 | 0.948 | 6.154 | -2.559 |
| 2_1_0_1_-1 | /A/E63/401// | 26.567 | 0.948 | 5.822 | -3.229 |
|  |  |  |  |  |  |
|  |  | **Syk Set** |  |  |  |
|  |  | 4YJQ |  |  |  |
| Species | Residue | StrainEnergy | RSCC | ZDD | XModeScore |
| 1_0_0_0_0 | /A/4DK/701// | 8.417 | 0.956 | 5.399 | 2.179 |
| 0_0_0_0_-1 | /A/4DK/701// | 9.141 | 0.954 | 5.714 | 1.242 |
| 5_1_0_0_1 | /A/4DK/701// | 13.148 | 0.955 | 5.373 | 0.675 |
| 2_1_0_0_0 | /A/4DK/701// | 8.617 | 0.951 | 6.438 | -0.19 |
| 6_1_1_0_1 | /A/4DK/701// | 13.086 | 0.95 | 6.456 | -1.704 |
| 4_0_0_0_1 | /A/4DK/701// | 16.645 | 0.951 | 6.15 | -2.201 |
|  |  |  |  |  |  |
|  |  | 4YJR |  |  |  |
| Species | Residue | StrainEnergy | RSCC | ZDD | XModeScore |
| 1_0_0_0_0 | /A/4DJ/701// | 6.567 | 0.958 | 7.154 | 2.947 |
| 0_0_0_0_-1 | /A/4DJ/701// | 9.193 | 0.956 | 7.55 | 0.477 |
| 5_1_0_0_1 | /A/4DJ/701// | 15.753 | 0.955 | 7.632 | -1.399 |
| 4_0_0_0_1 | /A/4DJ/701// | 17.139 | 0.956 | 7.698 | -2.025 |
|  |  |  |  |  |  |
|  |  |  |  |  |  |
|  |  | 4YJT |  |  |  |
| Species | Residue | StrainEnergy | RSCC | ZDD | XModeScore |
| 3_0_0_0_0 | /A/4DQ/702// | 7.578 | 0.956 | 7.722 | 2.202 |
| 1_1_0_0_-1 | /A/4DQ/702// | 12.498 | 0.959 | 7.422 | 0.93 |
| 5_1_1_0_0 | /A/4DQ/702// | 9.92 | 0.956 | 8.135 | 0.489 |
| 0_0_0_0_-1 | /A/4DQ/702// | 10.237 | 0.955 | 8.387 | -0.132 |
| 2_1_0_1_-1 | /A/4DQ/702// | 10.866 | 0.954 | 9.014 | -1.618 |
| 6_0_0_0_1 | /A/4DQ/702// | 16.112 | 0.952 | 8.14 | -1.871 |
|  |  |  |  |  |  |
|  |  | 4YJU |  |  |  |
| Species | Residue | StrainEnergy | RSCC | ZDD | XModeScore |
| 3_0_0_0_0 | /A/4DO/701// | 17.297 | 0.97 | 4.008 | 1.298 |
| 0_0_0_0_-1 | /A/4DO/701// | 16.028 | 0.968 | 4.098 | 1.198 |
| 6_0_0_0_1 | /A/4DO/701// | 32.282 | 0.967 | 3.994 | -0.986 |
| 2_1_0_1_-1 | /A/4DO/701// | 20.094 | 0.967 | 4.731 | -1.51 |
|  |  |  |  |  |  |
|  |  | 4YJV |  |  |  |
| Species | Residue | Strain | Energy | RSCC | ZDD |
| 0_0_0_0_-1 | /A/4DT/701// | 11.392 | 0.934 | 5.904 | 1.854 |
| 2_1_0_0_0 | /A/4DT/701// | 12.324 | 0.933 | 6.018 | 1.347 |
| 3_1_0_1_0 | /A/4DT/701// | 32.113 | 0.935 | 5.655 | 1.085 |
| 6_1_1_0_1 | /A/4DT/701// | 28.24 | 0.934 | 5.778 | 0.941 |
| 1_0_0_0_0 | /A/4DT/701// | 35.065 | 0.933 | 6.066 | -0.705 |
| 4_0_0_0_1 | /A/4DT/701// | 30.831 | 0.933 | 6.24 | -1.011 |
| 5_1_0_0_1 | /A/4DT/701// | 48.937 | 0.932 | 6.509 | -3.511 |
|  |  |  |  |  |  |
|  |  | **uPA Set** |  |  |  |
|  |  | 4FUD |  |  |  |
| Species | Residue | StrainEnergy | RSCC | ZDD | XModeScore |
| 3_0_0_0_0 | /A/6UP/301// | 4.304 | 0.944 | 2.857 | 2.18 |
| 1_1_0_0_-1 | /A/6UP/301// | 3.701 | 0.939 | 3.239 | -0.047 |
| 2_1_1_0_-1 | /A/6UP/301// | 6.016 | 0.943 | 3.182 | -0.967 |
| 0_0_0_0_-1 | /A/6UP/301// | 8.27 | 0.944 | 3.022 | -1.166 |
|  |  |  |  |  |  |
|  |  | 4FUE |  |  |  |
| Species | Residue | StrainEnergy | RSCC | ZDD | XModeScore |
| 1_0_0_0_0 | /A/7UP/301// | 7.49 | 0.941 | 3.157 | 1.164 |
| 6_1_1_0_1 | /A/7UP/301// | 9.91 | 0.937 | 3.086 | 0.374 |
| 5_1_0_0_1 | /A/7UP/301// | 10.089 | 0.942 | 3.092 | 0.178 |
| 0_0_0_0_-1 | /A/7UP/301// | 6.418 | 0.937 | 3.306 | 0.005 |
| 3_1_0_1_0 | /A/7UP/301// | 8.444 | 0.94 | 3.203 | -0.103 |
| 4_0_0_0_1 | /A/7UP/301// | 10.091 | 0.935 | 3.231 | -1.617 |
|  |  |  |  |  |  |
|  |  | 4FU7 |  |  |  |
| Species | Residue | StrainEnergy | RSCC | ZDD | XModeScore |
| 1_0_0_0_0 | /A/1UP/305// | 11.113 | 0.951 | 2.371 | 1.604 |
| 0_0_0_0_-1 | /A/1UP/305// | 10.205 | 0.949 | 2.747 | 0.638 |
| 3_0_0_0_1 | /A/1UP/305// | 16.191 | 0.951 | 2.523 | -1.113 |
| 2_1_0_0_0 | /A/1UP/305// | 11.292 | 0.947 | 3.109 | -1.13 |
|  |  |  |  |  |  |
|  |  | 4FU8 |  |  |  |
| Species | Residue | Strain | Energy | RSCC | ZDD |
| 3_0_0_0_1 | /A/2UP/301// | 3.567 | 0.962 | 2.598 | 1.985 |
| 2_1_0_0_0 | /A/2UP/301// | 4.554 | 0.963 | 2.794 | 0.752 |
| 1_0_0_0_0 | /A/2UP/301// | 4.05 | 0.96 | 2.924 | 0.53 |
| 0_0_0_0_-1 | /A/2UP/301// | 8.487 | 0.957 | 3.336 | -3.268 |
|  |  |  |  |  |  |
|  |  | 4FU9 |  |  |  |
| Species | Residue | StrainEnergy | RSCC | ZDD | XModeScore |
| 3_0_0_0_0 | /A/675/313// | 7.2 | 0.971 | 1.288 | 1.281 |
| 1_1_0_0_-1 | /A/675/313// | 5.462 | 0.97 | 1.552 | 1.147 |
| 2_1_1_0_-1 | /A/675/313// | 9.046 | 0.969 | 1.776 | -2.428 |
|  |  |  |  |  |  |
|  |  | 4FUB |  |  |  |
| Species | Residue | StrainEnergy | RSCC | ZDD | XModeScore |
| 1_0_0_0_0 | /A/4UP/301// | 6.807 | 0.966 | 1.785 | 1.004 |
| 0_0_0_0_-1 | /A/4UP/301// | 8.63 | 0.967 | 1.531 | 0.976 |
| 2_1_0_0_0 | /A/4UP/301// | 6.21 | 0.966 | 1.945 | 0.654 |
| 3_0_0_0_1 | /A/4UP/301// | 9.973 | 0.966 | 2.107 | -2.634 |
|  |  |  |  |  |  |
|  |  | 4FUC |  |  |  |
| Species | Residue | StrainEnergy | RSCC | ZDD | XModeScore |
| 0_0_0_0_-1 | /A/239/301// | 9.645 | 0.968 | 1.893 | 2 |
| 1_0_0_0_0 | /A/239/301// | 11.097 | 0.957 | 5.01 | -2 |
